# The genome and occlusion bodies of marine *Penaeus monodon* nudivirus (PmNV, also known as MBV and PemoNPV) suggest that it should be assigned to a new nudivirus genus that is distinct from the terrestrial nudiviruses

**DOI:** 10.1186/1471-2164-15-628

**Published:** 2014-07-25

**Authors:** Yi-Ting Yang, Der-Yen Lee, Yongjie Wang, Jer-Ming Hu, Wen-Hsiung Li, Jiann-Horng Leu, Geen-Dong Chang, Huei-Mien Ke, Shin-Ting Kang, Shih-Shun Lin, Guang-Hsiung Kou, Chu-Fang Lo

**Affiliations:** Institute of Bioinformatics and Biosignal Transduction, College of Bioscience and Biotechnology, National Cheng Kung University, Tainan, Taiwan; Department of Life Science, National Taiwan University, Taipei, Taiwan; Technology Commons, College of Life Science, National Taiwan University, Taipei, Taiwan; Laboratory of Quality and Safety Risk Assessment for Aquatic Products on Storage & Preservation (Shanghai), Ministry of Agriculture, Shanghai, China; College of Food Science and Technology, Shanghai Ocean University, Shanghai, China; Institute of Ecology and Evolutionary Biology, National Taiwan University, Taipei, Taiwan; Biodiversity Research Center, Academia Sinica, Taipei, Taiwan; Department of Ecology and Evolution, University of Chicago, Chicago, USA; Institute of Marine Biology, College of Life Sciences, National Taiwan Ocean University, Taipei, Taiwan; Center of Excellence for the Oceans, National Taiwan Ocean University, Keelung City, Taiwan; Institute of Biochemical Sciences, College of Life Science, National Taiwan University, Taipei, Taiwan; Ph.D. Program in Microbial Genomics, National Chung Hsing University and Academia Sinica, Taipei, Taiwan; Institute of Biotechnology, National Taiwan University, Taipei, Taiwan; Center of Bioscience and Biotechnology, National Cheng Kung University, Tainan, Taiwan

**Keywords:** PmNV, Genome, Baculovirus, Nudivirus, OBs, Polyhedrin

## Abstract

**Background:**

*Penaeus monodon* nudivirus (PmNV) is the causative agent of spherical baculovirosis in shrimp (*Penaeus monodon*). This disease causes significant mortalities at the larval stage and early postlarval (PL) stage and may suppress growth and reduce survival and production in aquaculture. The nomenclature and classification status of PmNV has been changed several times due to morphological observation and phylogenetic analysis of its partial genome sequence. In this study, we therefore completed the genome sequence and constructed phylogenetic trees to clarify PmNV’s taxonomic position. To better understand the characteristics of the occlusion bodies formed by this marine occluded virus, we also compared the chemical properties of the polyhedrin produced by PmNV and the baculovirus AcMNPV (*Autographa californica* nucleopolyhedrovirus).

**Results:**

We used next generation sequencing and traditional PCR methods to obtain the complete PmNV genome sequence of 119,638 bp encoding 115 putative ORFs. Phylogenetic tree analysis showed that several PmNV genes and sequences clustered with the non-occluded nudiviruses and not with the baculoviruses. We also investigated the characteristics of PmNV polyhedrin, which is a functionally important protein and the major component of the viral OBs (occlusion bodies). We found that both recombinant PmNV polyhedrin and wild-type PmNV OBs were sensitive to acid conditions, but unlike the baculoviral OBs, they were not susceptible to alkali treatment.

**Conclusions:**

From the viral genome features and phylogenetic analysis we conclude that PmNV is not a baculovirus, and that it should be assigned to the proposed *Nudiviridae* family with the other nudiviruses, but into a distinct new genus (*Gammanudivirus*).

**Electronic supplementary material:**

The online version of this article (doi:10.1186/1471-2164-15-628) contains supplementary material, which is available to authorized users.

## Background

Spherical baculovirosis is a shrimp disease that was first observed in Taiwan, and it was also the first reported viral disease from *Penaeus monodon* [1] *.* The viral pathogen that causes this disease is now widely distributed along the Indo-Pacific coasts of Asia, and it infects a range of penaeid shrimps. The virus is a rod-shaped, singly enveloped and occluded large circular dsDNA virus that replicates within the nucleus, and it targets several organs, including the hepatopancreatic tubule epithelium and duct epithelium of postlarvae, juveniles and adults, and the anterior midgut epithelium of very young postlarvae [2].

When it was first discovered in 1981, it was thought to be a baculovirus because of the structure of its occlusion bodies as revealed by electron microscopy [1]. Two years later, it was designated monodon baculovirus (MBV) [2], and this name is still commonly used today. One decade after its discovery, Mari *et al*. showed that each envelope contained a single nucleocapsid, and that numerous virions were included within each occlusion body. Mari *et al*. therefore proposed that MBV be assigned to the subgenus SNPV with the name PmSNPV [3]. Later still, from 2005 to 2011, in the 8^th^ report of the International Committee on Taxonomy of Viruses (ICTV), *Penaeus monodon* nucleopolyhedrovirus (PemoNPV) was listed as a “tentative species” in the *Nucleopolyhedrovirus* genus. Meanwhile, even though the virus forms occlusion bodies, in 2009, Wang and Jehle used a molecular phylogenetic analysis of six viral genes and supermatrix methods to propose that this virus should to be re-assigned to the genus *Nudivirus*. They also proposed that it be renamed to *P. monodon* nudivirus, PmNV [4]. Although the taxonomic status of this virus is still in dispute, and it is not included in the 9^th^ ICTV report (2012), the evidence we present here supports this proposed reassignment, and we will use this new name throughout the present manuscript.

Before the present study, only a 22.8 Kb partial PmNV genome sequence was available in GenBank. To further clarify its phylogenetic status, we used an NGS (Next Generation Sequencing) platform to determine the complete genome sequence of PmNV, and then analyzed and compared its genomic features with other closely related species. We also investigated the unique properties of the polyhedrin that forms PmNV’s occlusion bodies. Our results suggest that PmNV is not a baculovirus, and we propose that it should be assigned to a third genus, *Gammanudivirus*, within the newly proposed *Nudiviridae* family [5].

## Results and discussion

### Sequencing of the PmNV genome

The complete PmNV genome was sequenced by an Illumina Miseq sequencer using the paired-end method. High-throughput sequencing was performed twice to compare between different sample preparation methods and sequencing conditions. The longest contigs from the first and the second sequencing were 119,426 and 119,128 nt, respectively. However, high-throughput NGS sequencing can produce nucleotide errors, while the short sequences used by NGS can lead to repetitive errors in assembly. Therefore, based on the longest contig, we designed 374 primers (Additional file 1: Table S1) and used Sanger sequencing to recheck sequences that had unreliable signals. A total of 754 runs were assembled into a single contiguous sequence with a size of 119,638 bp. Comparisons of this sequence with the two high-throughput contigs found a 1 nt insertion and a total of 211 nt deletions in the contig from the first high-throughput sequencing, and 3 nucleotide errors and 556 nt deletions in the contig from the second high-throughput sequencing (Additional file 2: Table S2).

Although the second sequencing produced about 3 × more data than the first, its mapping rate was about 3 × lower, and overall, the mappable reads were almost the same (Additional file 3: Table S3). Since these two high-throughput sequencings were performed on samples of viral genomic DNA with different purity and concentration, the similarity of the two sequencing results therefore suggests that good results can be obtained even when the sample is contaminated with host DNA as long as there is sufficient coverage for accurate assembly.

We conclude that the complete circular genome of PmNV is 119,638 bp in size. This length is in good agreement with the 80-160 kb estimated by *Bam*HI digestion [3]. The PmNV genome has a G + C content of 34.5%, which is lower than the G + C content of the type species of the baculoviruses (AcMNPV) and all but one of the four nudiviruses (Table 1).

**Table 1:** **Comparisons of the genome of PmNV and various dsDNA virus**

Virus	Genome size (bp)	No. of ORF	G + C content (%)	Gene density (kb)	Reference
PmNV	119,638	115	34.5	1.04	This study
HzNV-1	228,108	155	41.8	1.47	Cheng *et al.* [6]
HzNV-2	231,621	113	42.0	2.04	Burand *et al.* [7]
GbNV	96,944	98	28.0	0.99	Wang *et al.* [8]
OrNV	127,615	139	42.0	0.92	Wang *et al.,* [9]
AcMNPV	133,894	156	40.0	0.73	Ayres *et al.,* [10]

### Repetitive sequences in the PmNV genome

**Figure 1:**
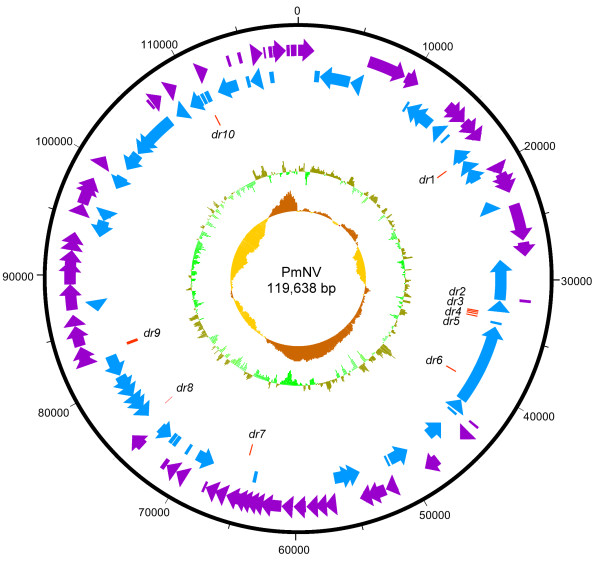
**Circular map of the PmNV genome.** Purple represents the 60 forward strand ORFs and blue represents the 55 reverse strand ORFs. Red represents the 10 direct repeat (*dr*) regions, which are dispersed around the genome. The innermost circle shows GC skew, which indicates possible locations of the DNA leading strand, lagging strand, replication origin, and replication terminal during DNA replication. Below average GC skew is light orange and above average dark orange. The next innermost circle is a GC plot, with light green representing below average GC content, and dark green indicating above average GC content.

Homology regions (*hrs*) are an important feature in the genomes of many dsDNA viruses. These regions, which are AT-rich and consist of direct repeats with imperfect palindrome sequences, play a central role in replication [11–13] and also act as transcriptional enhancers [14]. *hrs* vary in length, sequence and copy number between species, and most of the baculoviruses have several *hrs* distributed around their respective genomes. Notable exceptions include *Trichoplusia ni* single nucleopolyhedrovirus (TnSNPV) [15], *Chrysodeixis chalcites* nucleopolyhedrovirus (ChchNPV) [16] and *Agrotis segetum* granuloviruses (AgseGV) [17], none of which have any *hrs*. To date, no *hrs* have been found in any of the fully sequenced nudiviruses, ie. HzNV-1 (*Heliothis zea* nudivirus 1) [6], HzNV-2 (*Helicoverpa zea* nudivirus 2) [7], GbNV (*Gryllus bimaculatus* nudivirus) [8] and OrNV (*Oryctes rhinoceros* nudivirus) [18]. The PmNV genome also does not include any *hrs*. Like the nudiviruses, however, it does include a number of tandem direct repeats (*dr*; Additional file 4: Table S5). Four of these repeats (*dr*2-5) cluster together between locations 33,271 and 33,962 in the PmNV genome, which is predicted to be a non-coding region (Figure 1). The repeat unit, copy number and total length of the repeats range from 3 to 42 bp, 2.0 to 8.7 and 26 to 284 bp, respectively, which means that repeat sequences are less abundant in PmNV than in, for example, HzNV-2. Taken together, these results suggest that PmNV and the nudiviruses might have a replication origin pattern which is distinct from the baculoviruses.

### ORF prediction

The ORFs predicted by DNAMAN and the online programs GeneMarkS, GLIMMER3 and FGENESV0 are shown in Additional file 5: Table S4. The A of the ATG start codon of the PmNV001 (*polyhedrin* gene [19]) was defined as nucleotide 1 of the PmNV genome. To reduce the number of false positives and improve accuracy, only those ORFs that were predicted by at least two programs or which had an E-value of less than 1 in the BLASTP search were selected for further analysis. In total, 60 predicted ORFs were found on the forward strand and 55 predicted ORFs on the reverse strand, with sizes ranging from 144 bp to 7.5 kb (Figure 1). The average length of the predicted ORFs was about 990 bp and the gene density was 1.04 per kb. Eight of the 115 putative ORFs (PmNV003, PmNV006, PmNV016, PmNV033, PmNV049, PmNV067, PmNV073 and PmNV113) are similar to proteins found in eukaryotic organisms, while 45 ORFs (39% of 115 putative ORFs) have significant homologies to genes found in other dsDNA viruses (Table 2). Homologous genes were found especially in two closely related nudiviruses with 93% sequence identity, HzNV-1 and HzNV-2 [7]. By comparison, the *Baculoviridae* share 37 core genes among the *Alphabaculovirus, Betabaculovirus, Gammabaculovirus* and *Deltabaculovirus* [20], 20 of which are also homologous to nudivirus genes [18]. Except for *vp39,* PmNV has the same homologous genes as the nudiviruses (Table 2). In addition to the nudiviruses and baculoviruses, PmNV also shares some homologous genes with the bracoviruses and hytrosaviruses, two viral groups that are closely related to the nudiviruses and baculoviruses (Table 3).

**Table 2:** **Cross reference of the 37 conserved and 4 other baculovirus genes from AcMNPV with their homologs in the nudiviruses (OrNV, GbNV, HzNV-1 and HzNV-2) and PmNV**

Function	Gene name	***Nudivirus***					PmNV ORF/aa	Bracoviruses and hytrosaviruses			
		ORF (aa identity/similarity%)						Accecssion no./ORF (aa identity/similarity%)			
		AcMNPV	OrNV	GbNV	HzNV-1	HzNV-2		CcBV	CiBV	MdSGHV	GpSGHV
**37 baculovirus core genes**											
**Replication**	*dnapol*	65 (8/24)	1 (12/30)	12 (16/36)	131 (24/46)	18 (24/46)	5/1091	NA	NA	1 (8/22)	79 (9/24)
	*helicase*	95 (9/26)	34 (12/31)	88 (11/28)	104 (16/31)	38 (16/31)	94/1271	NA	NA	-	-
	*lef-1*	14	-	-	-	-	-	NA	NA	-	-
	*lef-2*	6	-	-	-	-	-	NA	NA	-	-
	*alk-exo*	133	-	-	-	-	-	NA	NA	-	-
**Transcription**	*p47*	40 (8/20)	20 (14/26)	69 (10/21)	*75 (7/15)	*63 (7/15)	14/419	CAR31573 (17/33)	NA	-	-
	*lef-4*	90 (8/21)	42 (15/31)	96 (14/33)	98 (10/19)	43 (10/19)	91/470	NA	CAR40187 (6/14)	-	-
	*lef-5*	99 (4/17)	52 (9/16)	85 (9/12)	101 (14/23)	40 (14/23)	52/175	CAT00573 (12/27)	NA	-	-
	*lef-8*	50 (9/24)	64 (17/34)	49 (18/35)	90 (17/33)	51 (17/33)	23/1032	CAR82252 (18/33)	CBB83982 (3/6)	-	-
	*lef-9*	62 (9/27)	96 (15/32)	24 (16/32)	*75 (12/21)	*63 (12/21)	58/574	NA	NA	-	-
**Oral infectivity**	*pif-0 (p74)*	138 (17/38)	126 (24/43)	45 (23/43)	11 (26/48)	106 (26/47)	72/684	CAR82260 (24/45)	CAR40192(24/45)	39 (13/28)	1 (12/30)
	*pif-1*	119 (11/25)	60 (20/34)	52 (18/32)	55 (22/38)	82 (22/38)	39/525	NA	NA	29 (9/20)	102 (10/21)
	*pif-2*	22 (14/27)	17 (20/35)	66 (20/34)	123 (25/43)	26 (25/43)	15/430	NA	CAR40194 (17/32)	89 (12/26)	53 (20/34)
	*pif-3*	115 (14/23)	107 (22/38)	3 (24/40)	88 (28/43)	53 (28/43)	93/192	CAR82247 (20/38)	NA	106 (14/23)	101 (5/12)
	*pif-4 (19 k/odv-e28)*	96 (14/31)	33 (20/35)	87 (17/31)	103 (9/16)	39 (9/16)	96/232	CAR31579 (17/36)	CAR40196 (21/38)	-	-
	*pif-5 (odv-e56)*	148 (10/24)	115 (14/32)	5 (16/34)	76 (22/42)	62 (21/42)	10/449	CAR31578 (15/33)	CAR31577 (14/30)	-	97 (11/25)
	*pif-6 (ac68)*	68 (9/25)	72 (19/42)	55 (20/43)	74 (20/41)	64 (20/40)	88/143	CAR82241 (9/25)	NA	-	-
**Packaging, assembly, and release**	*38 k*	98 (10/27)	87 (14/30)	1 (13/27)	10 (21/38)	108 (21/38)	59/282	CAR82239 (16/33)	CAR40188 (19/38)	-	-
	*p6.9*	100	-	-	-	-	-	NA	NA	-	-
	**vlf-1	77 (6/18)	30 (6/15)	80 (12/26)	121 (10/22)	28 (10/22)	56/289	CAR40203 (16/35)	CAR40190 (13/29)	-	-
	**vlf-1	77 (5/18)	30 (4/10)	80 (9/26)	121 (8/22)	28 (8/22)	90/240	CAR40203 (19/35)	CAR40190 (17/33)	-	-
	*vp39*	89	15	64	89	52	-	NA	NA	-	-
	*vp1054*	54	-	-	-	-	-	NA	NA	-	-
	*vp91/p95*	83 (10/23)	106 (17/34 )	2 (14/29)	46 (17/34)	89 (17/34)	9/675	NA	NA	-	-
	*gp41*	80	-	-	-	-	-	NA	NA	-	-
	*p6.9*	100	-	-	-	-	-	NA	NA	-	-
	*p18*	93	-	-	-	-	-	NA	NA	-	-
	*p33*	92 (9/25)	113 (11/20)	7 (11/23)	13 (24/42)	104 (25/42)	8/228	NA	NA	-	-
	*p40*	101	-	-	-	-	-	NA	NA	-	-
	*p48*	103	-	-	-	-	-	NA	NA	-	-
	*p49*	142	-	-	-	-	-	NA	NA	-	-
	*odv-ec43*	109	-	-	-	-	-	NA	NA	-	-
	*odv-e18*	143	-	-	-	-	-	NA	NA	-	-
	*odv-e27*	144	-	-	-	-	-	NA	NA	-	-
	*desmoplakin*	66	-	-	-	-	-	NA	NA	-	-
	*ac53*	53	-	-	-	-	-	NA	NA	-	-
**Unknown function**	*ac81*	81 (10/21)	4 (23/41)	14 (20/41)	33 (32/54)	96 (33/54)	86/157	NA	NA	108 (13/26)	-
	*ac78*	78	-	-	-	-	-	NA	NA	-	-
**Other baculovirus genes**											
**Transferase enzyme**	*methyltransferase*	69 (14/24)	-	-	37 (7/14)	93 (7/14)	6/401	NA	NA	-	-
**ODV envelope**	**odv-e66	46 (10/20)	12 (16/32)	-	-	-	34/551	NA	NA	47 (12/23)	5 (8/14)
	**odv-e66	46 (12/24)	12 (15/30)	-	-	-	36/635	NA	NA	47 (13/28)	5 (9/16)
**Anti-apoptosis**	*iap*	27 (6/12)	134 (7/11)	98 (7/14)	135 (14/23)	15 (14/23)	106/101	NA	NA	78 (13/24)	-
**Occlusion body**	*polyhedrin*	8 (5/15)	16 (11/25)	65 (12/27)	68 (7/20)	70 (7/20)	^#^20/423	NA	NA	76(10/23)	-
	*polyhedrin*	8	-	-	-	-	^##^1/452	-	-	-	-

^*^In HzNV-1 both fused in the same ORF and appear to be fused into a single gene.

^**^The PmNV genome contains two ORFs that are homologous to *vlf-1* and to *odv-e66*.

^#^Despite the [low] identity and similarity between PmNV020 and other polyhedrons from AcMNPV and the nudiviruses, this ORF does not in fact seem to express a functional polyhedrin.

^##^PmNV001 is a functional homolog of *polyhedrin* that shows no sequence homology to AcMNPV *polyhedrin*.

Bracoviruses and hytrosaviruses are included for comparison.

**Table 3:** **Cross reference of PmNV genes that have homologs in the nudiviruses (OrNV, GbNV, HzNV-1 and HzNV-2), bracoviruses (CcBV and CiBV), hytrosaviruses (MdSGHV and GpSGHV)**

Function	Gene name	***Nudivirus***				ORF/aa PmNV	Bracoviruses and hytrosaviruses			
		ORF (aa% identity/similarity)					Accecssion no./ORF (aa% identity/similarity)			
		OrNV	GbNV	HzNV-1	HzNV-2		CcBV	CiBV	MdSGHV	GpSGHV
**Replication**	*DNA excision and repair*	16 (11/25)	65 (12/25)	68 (9/19)	70 (9/19)	20/423	NA	NA	76 (12/25)	-
	*integrase*	75 (20/39)	57 (21/39)	144 (25/43)	8 (25/43)	55/306	CAR40240 (21/39)	NA	-	-
	*helicase 2	108 (5/18)	46 (8/25)	60 (16/32)	76 (16/32)	76/576	NA	NA	104 (6/19)	074 (7/23)
	*helicase 2	108 (6/20)	46 (8/22)	60 (14/30)	76 (14/30)	79/715	NA	NA	104 (6/19)	074 (7/23)
**Structural protein**	*PmV-like*	-	-	118 (9/22)	30 (9/24)	45/248	CAR31589 (15/29)	CAR40204 (15/32)	100 (8/17)	-
	**HzNVORF106-like	3 (7/21)	13 (9/25)	106 (14/30)	37 (9/25)	69/328	CAR31586 (12/30)	CAR40201 (14/29)	-	-
	**HzNVORF140-like	-	-	140 (14/30)	11 (7/14)	90/240	CAR31588 (17/37)	CAR40203 (6/13)	-	-
	**HzNVORF9-like	-	-	9 (14/30)	109 (20/41)	107/235	CAR31582 (21/44)	CAR40198 (18/42)	-	-
**Other known and unknown functions**	*31 K structural protein*	-	-	89 (13/31)	52 (13/31)	22/310	CAR31585 (17/33)	CAR40200 (18/32)	-	-
	*p51*	-	-	64 (15/38)	73 (15/38)	24/397	CAR31584 (16/39)	NA	-	-
	*Guanosine monophosphate kinase*	117 (12/28)	34 (12/31)	111 (19/40)	34 (19/40)	38/314	NA	NA	-	-
	**HzNVORF128-like	-	-	128 (6/22)	21 (6/22)	42/616	CAR31587 (7/21)	CAR40202 (3/9)	-	-
	*thymidylate kinase*	125 (12/22)	44 (15/31)	115 (14/30)	32 (10/25)	46/338	NA	NA	-	-
	*DNA ligase-like*	131 (3/9)	74 (5/15)	141 (14/30)	10 (14/30)	48/590	NA	NA	-	-
	*orf9* (HzNV2)	76 (32/58)	58 (10/30)	143 (14/30)	9 (29/57)	53/54	NA	NA	-	-
	*thymidylate kinase*	137 (14/27)	17 (14/29)	51 (16/31)	85 (16/33)	65/437	NA	NA	-	-
	*orf84* (HzNV-2)	-	-	52 (14/30)	84 (6/12)	66/416	NA	NA	-	-
	**HzNVORF100	-	-	100 (14/30)	41 (15/34)	78/219	NA	NA	-	-
	*esterase/lipase*	47 (16/30)	19 (12/30)	30 (17/38)	99 (17/38)	98/277	NA	NA	-	-
	*microtubule-associated-like*	18 (11/29)	67 (14/31)	122 (14/30)	27 (13/30)	99/414	NA	NA	-	-
	*11 K virion structural protein*	41 (30/46)	95 (13/35)	124 (18/42)	25 (18/42)	100/101	NA	NA	-	-
	**HzNVORF28-like	-	-	28 (14/30)	101 (14/28)	108/243	NA	NA	-	-

*The PmNV genome contains two ORFs that are homologous to *helicase 2.*

**“HzNVORF” genes use the HzNV-1 ORF number.

Identity and similarity values in Tables 2 and 3 were calculated by ClustalX2 and GeneDoc and are different from the BLASTP results in Table 4 and in the text.

### Gene parity analysis

Gene parity plots that compared the gene organization of PmNV’s predicted ORFs with those of two representative nudivirus species, HzNV-1 and OrNV, showed no significantly similar pattern of gene location within the respective genomes (Additional file 6: FigureS1). However, gene parity is not generally conserved in either baculoviruses or nudiviruses, and we note that the conserved gene cluster of *helicase* and *pif-4* (19 kDa) that is found in the known nudiviruses [18] was in fact also detected in the PmNV genome (PmNV094 and PmNV096) though in opposite orientation (Additional file 7: FigureS2). This suggests that PmNV has a closer evolutionary relationship to the nudiviruses than to other large dsDNA viruses.

### Functional and phylogenetic analysis of PmNV putative ORFs that are homologous to baculovirus (AcMNPV) genes

#### Enzymes involved in DNA replication

The PmNV genome includes two ORFs, PmNV005 (*dna polymerase*) and PmNV094 (*helicase*), that are involved in DNA replication and play essential roles in DNA polymerization and DNA unwinding, respectively. Two other enzymes LEF-1 and LEF-2, which are factors essential for primase activity in baculoviruses, did not have any homologs in PmNV.

**Figure 2:**
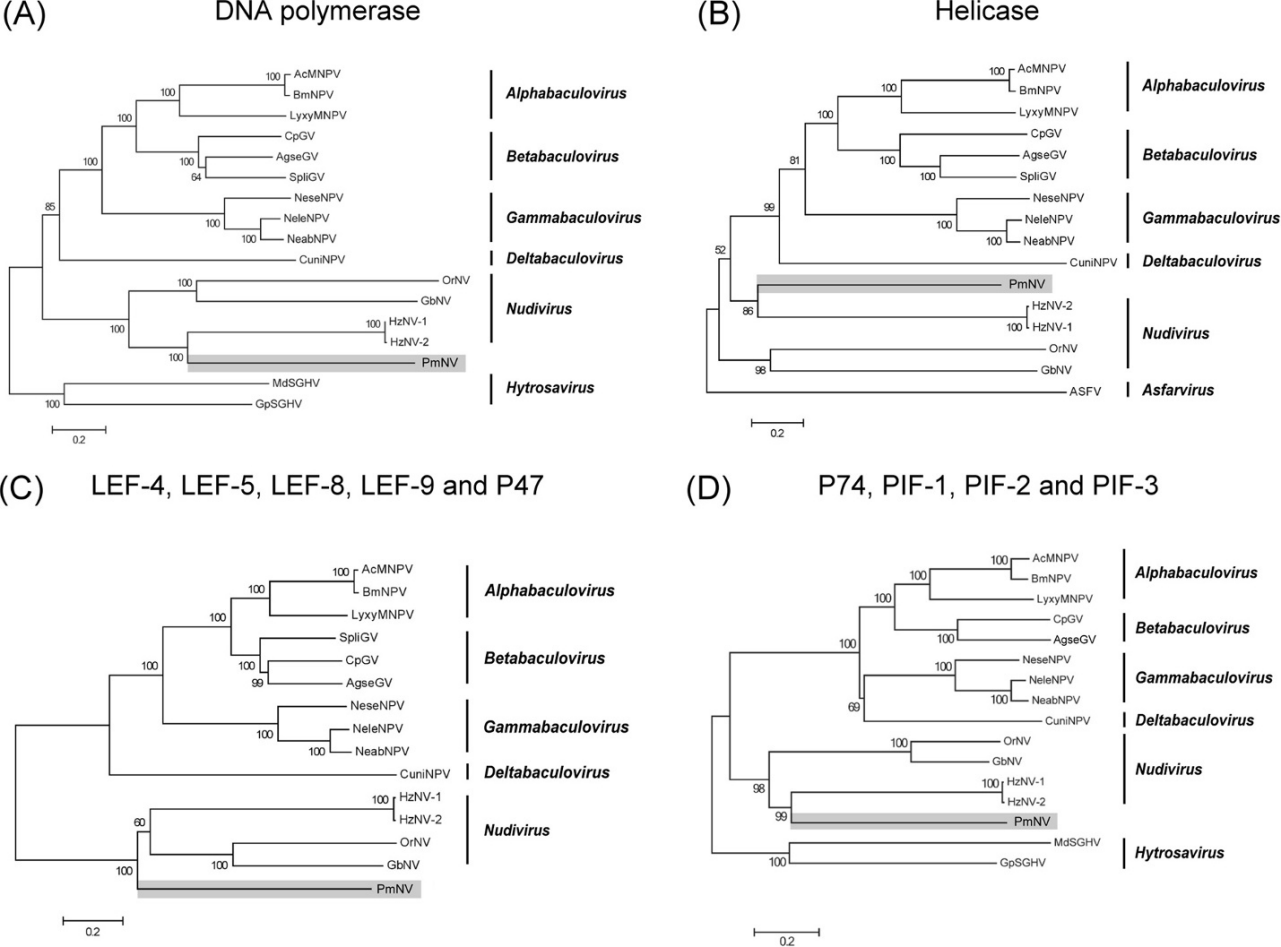
**Phylogenetic analysis of selected DNA viruses and PmNV.** Multiple alignments of **(A)** DNA polymerase*,* **(B)** Helicase, **(C)** LEF-4, LEF-5, LEF-8, LEF-9 and P47 **(D)** P74, PIF-1, PIF-2 and PIF-3 protein sequences were performed using BioEdit. The trees were inferred using MEGA5.2 and the neighbor-joining method. The robustness of each tree was tested using bootstrap (1000) analysis. The percent values are indicated at the nodes. Organisms included in this analysis, with abbreviated names: *Autographa californica* multiple nucleopolyhedrovirus (AcMNPV), *Bombyx mori* nucleopolyhedrovirus (BmNPV), *Lymantria xylin* nucleopolyhedrovirus (LyxyMNPV), *Cydia prmonella* granulovirus (CpGV), *Agrotis segetum* granulovirus (AgseGV), *Spodoptera litura* granulovirus (SpliGV), *Neodiprion sertifer* nucleopolyhedrovirus (NeseNPV), *Neodiprion lecontei* nucleopolyhedrovirus (NeleNPV), *Neodiprion Abietis* nucleopolyhedrovirus (NeabNPV), *Culex nigripalpus* nucleopolyhedrovirus (CuniNPV), *Heliothis zea* virus 1 (HzNV-1), *Helicoverpa zea* nudivirus 2 (HzNV-2), *Oryctes rhinoceros* nudivirus (OrNV), *Gryllus bimaculatus* nudivirus (GbNV), *Musca domestica* salivary gland hypertrophy virus (MdSGHV), *Glossina pallidipes* salivary gland hypertrophy virus (MdSGHV), African swine fever virus (ASFV). Accession numbers of all the sequences are listed in Additional file 8: Table S6.

PmNV005 was predicted to belong to the DNA polymerase type-B family, and it has the POLBc signature from 151-304 aa (Pfam: E = 1.5 × 10^-19^). The closest matches for PmNV005 are the DNA polymerases of HzNV-1 and HzNV-2, which both have 26% amino acid identity (BLASTP: E = 2 × 10^-102^; Table 4). DNA polymerase is a key enzyme in virus taxonomy, and it has often been used to construct phylogenetic trees of DNA viruses. In Figure 2A, the putative PmNV DNA polymerase protein sequence (PmNV005) was aligned with DNA polymerase sequences from closely related dsDNA viruses, including baculoviruses, nudiviruses and hytrosaviruses (as an outgroup), and subjected to phylogenetic analysis. Each node had a high bootstrap support percentage, indicating that this tree was reliable. PmNV was on a branch that was distant from the baculoviruses and closely related to the nudiviruses, which is consistent with the result reported by Wang and Jehle [4] for the partial PmNV genome sequence. Except for HzNV-1 and HzNV-2, the tree lengths show that there are greater evolutionary distances between the nudiviruses than between the baculoviruses.

**Table 4:** **Annotation of PmNV putative ORFs**

ORF	Strand	Position		Length		Best BLAST match				Signature
		Start	End	nt	aa	ORF, protein encoded, or mass	Species	aa identity (% match identity)	E value	
1	+	1	1359	1356	452	polyhedrin				
2	-	1525	1989	462	154					coiled-coil
3	-	2033	4861	2826	942	von Willebrand factor type A	Frankia sp. CN3	33	8 × 10^-3^	vWFA, ZF_RING_2
4	-	4954	5913	957	319					
5	+	5998	9273	3273	1091	DNA polymerase	Helicoverpa zea nudivirus 2	26	2 × 10^-102^	POLBc
6	+	9354	10559	1203	401	methyltransferase	Ceratitis capitata	33		FtsJ
7	-	10563	10772	207	69					TM
8	-	10779	11455	684	228	Ac92-like (sulfhydryl oxidase)	Heliothis zea virus 1	42	1 × 10^-17^	Evr1_Alr, TM
9	-	11455	13482	2025	675	vp91 (capsid)	Oryctes rhinoceros virus	31	7 × 10^-32^	ChtBD2, TM
10	+	13622	14971	1347	449	Odv-e56/pif-5 (envelope)	Heliothis zea virus 1	27	2 × 10^-30^	TM
11	-	13780	14331	549	183					TM
12	+	15057	15536	477	159					
13	-	15325	15549	222	74					TM
14	+	15548	16807	1257	419	p47	Heliothis zea virus 1	24	1 × 10^-5^	
15	-	16816	18108	1290	430	per-os infectivity factor 2 (pif-2)	Macrobrachium nudivirus	66	2 × 10^-75^	Baculo_44, coiled-coil, TM
16	+	17191	17598	405	135	OHCU decarboxylase	Sarcophilus harrisii	27	3 × 10^-3^	TM
17	-	18139	18987	846	282	HZV_115-like	Oryctes rhinoceros virus	33	0.58	p-loop_NTPase, NK superfamily
18	-	19071	20372	1299	433					
19	+	20465	20869	402	134					
20	+	20812	22083	1269	423	flap endonuclease 1 (FEN-1)	Heliothis zea virus 1	29	3 × 10^-14^	
21	+	22080	22556	474	158					
22	-	22553	23485	930	310	31 K structural protein	Chelonus inanitus	24	1 × 10^-9^	
23	+	23615	26713	3096	1032	RNA polymerase (lef-8)	Heliothis zea virus 1	28	8 × 10^-53^	RNA_pol_Rpb2_6
24	+	26864	28057	1191	397	p51 late protein	Heliothis zea virus 1	23	4 × 10^-9^	SBP_BACTERIAL_1
25	-	28160	31906	3744	1248					AF-4, ZnF_C2H2
26	+	31688	31912	222	74					TM
27	-	31969	32715	744	248					
28	-	34005	34214	210	69					
29	-	34463	42040	7572	2524					TM
30	-	42077	42649	570	190					
31	+	43013	43204	189	63					
32	-	43300	43512	210	70					
33	+	43884	44783	897	299	tripartite motif-containing protein 10-like	Alligator mississippiensis	37	7 × 10^-9^	RING, coiled-coil
34	-	44939	46594	1653	551	odv-e66 (chondroitin lyase)	Oryctes rhinoceros virus	29	1 × 10^-23^	odv-e66
35	+	47292	48599	1305	435					
36	-	48994	50901	1905	635	odv-e66 (chondroitin lyase)	Oryctes rhinoceros virus	26	5 × 10^-22^	odv-e66, TM
37	-	51077	51280	201	67					
38	+	51353	52297	942	314	Guanosine monophosphate kinase	Heliothis zea virus 1	31	5 × 10^-17^	
39	+	52359	53936	1575	525	per-os infectivity factor 1 (pif-1)	Heliothis zea virus 1	35	5 × 10^-57^	PIF, TM
40	-	53953	54573	618	206					
41	+	54167	54595	426	142					TM
42	-	54573	56423	1848	616	orf21	Helicoverpa zea nudivirus 2	25	1 × 10^-7^	
43	+	56796	57572	774	258					
44	+	57636	58376	738	246					
45	+	58384	59130	744	248	histidine decarboxylase (PmV-like)	Paramecium bursaria Chlorella virus NY-2B	27	0.15	
46	+	59185	60201	1014	338	thymidylate kinase	Heliothis zea virus 1	27	2 × 10^-14^	p-loop_NTPase
47	+	60297	61211	912	304					
48	+	61235	63007	1770	590	DNA ligase-like	Helicoverpa zea nudivirus 2	24	2 × 10^-6^	
49	+	63022	63729	705	235	SJCHGC06586 protein	Schistosoma japonicum	38	1 × 10^-7^	RING, coiled-coil
50	-	63726	64037	309	103					
51	+	64012	64221	207	69					TM
52	+	64249	64776	525	175	late expression factor 5 (lef-5)	Spodoptera frugiperda MNPV	31	0.028	Baculo_LEF-5_C
53	+	65083	65247	162	54	orf9	Helicoverpa zea nudivirus 2	37	0.003	TM
54	+	65268	65930	660	220					
55	+	65979	66899	918	306	integrase	Helicoverpa zea nudivirus 2	33	1 × 10^-32^	INT_REC_C
56	+	66883	67752	867	289	very late expression factor 1	Heliothis zea virus 1	32	5 × 10^-11^	DNA_REC_C
57	-	67753	67959	204	68					coiled-coil
58	-	67965	69689	1722	574	lef-9	Heliothis zea virus1	32	3 × 10^-63^	
59	+	69705	70553	846	282	38 K protein	Chelonus inanitus	37	2 × 10^-17^	
60	-	70543	70824	279	93					
61	+	70823	71515	690	230	orf49	Helicoverpa zea nudivirus 2	29	2.9	
62	+	71512	71862	348	116					TM
63	-	71859	72215	354	118					
64	-	72286	72633	345	115					TM
65	-	72717	74030	1311	437	orf51	Heliothis zea virus1	30	7 × 10^-30^	p-loop_NTPase
66	+	74079	75329	1248	416	orf84	Helicoverpa zea nudivirus 2	23	9 × 10^-4^	
67	-	75597	76169	573	191		Crocosphaera watsonii	25	1 × 10^-4^	
68	-	76400	76963	561	187					
69	-	76980	77966	984	328	HzNVORF106-like protein	Cotesia congregata	24	1 × 10^-6^	
70	-	77935	78555	618	206					
71	-	78633	80174	1539	513					
72	-	80247	82301	2052	684	p74 / pif-0 (envelope)	Heliothis zea virus 1	30	4 × 10^-95^	Baculo_74, TM
73	+	82393	83277	882	294	Zonadhesin	Sorex araneus	49	2 × 10^-52^	TM
74	-	82986	83258	237	90					TM
75	+	83362	83934	570	190					
76	+	84006	85736	1728	576	helicase 2	Danaus plexippus	30	1 × 10^-46^	
77	+	85788	86687	897	299					
78	-	86652	87311	657	219	orf100	Heliothis zea virus 1	23	0.014	
79	+	87109	89256	2145	715	helicase 2	Danaus plexippus	28	5 × 10^-39^	TM
80	+	89240	92104	2862	954					coiled-coil
81	-	90949	91242	291	97					
82	+	92108	93043	933	311					
83	+	93036	93647	609	203					
84	-	93644	95206	1560	520					
85	-	95208	95618	408	136					
86	+	95617	96090	471	157	Ac81-like	Helicoverpa zea nudivirus 2	36	3 × 10^-21^	TM
87	+	96083	97993	1908	636					
88	+	98006	98437	429	143	Ac68-like/pif-6	Helicoverpa zea nudivirus 2	29	8 × 10^-11^	TM
89	-	98438	99691	1251	417					
90	+	99781	100530	720	240	VLF-1 protein	Helicoverpa zea nudivirus 2	28	5 × 10^-5^	
91	-	100500	101912	1410	470	late expression factor 4	Helicoverpa zea nudivirus 2	26	4 × 10^-18^	
92	-	101932	102255	321	107					
93	-	102312	102890	576	192	per-os infectivity factor 3	Helicoverpa zea nudivirus 2	38	4 × 10^-26^	TM
94	-	102887	106702	3813	1271	helicase	Helicoverpa zea nudivirus 2	28	3 × 10^-19^	primase_Cte
95	+	106149	106370	219	73					
96	+	106701	107399	696	232	19kda protein/Odv-e28/pif-4	Oryctes rhinoceros virus	38	5 × 10^-21^	Baculo_19, TM
97	-	107374	108123	747	249					
98	+	108113	108946	831	277	Esterase/lipase	Helicoverpa zea nudivirus 2	27	3 × 10^-4^	AAA_17, TM
99	-	108943	110187	1242	414	GrBNV_gp67-like	Oryctes rhinoceros virus	29	9 × 10^-8^	EF_HAND_1, coiled-coil
100	-	110290	110595	303	101	11 K virion structural protein	Oryctes rhinoceros virus	32	4 × 10^-6^	TONB_DEPENDENT_REC_1, TM
101	-	110705	111106	399	133					
102	+	111081	111881	798	266					
103	-	111883	113868	1983	661					
104	+	113631	113864	231	77					
105	+	114593	114865	270	90					
106	-	114757	115062	303	101	apoptosis inhibitor IAP-1	Buzura suppressaria NPV	43	2 × 10^-13^	BIR
107	-	115114	115821	705	235	HzNVORF9-like	Chelonus inanitus	25	4 × 10^-10^	
108	+	115917	116648	729	243	orf101	Helicoverpa zea nudivirus 2	23	0.1	ribokinase_pfkB_like, TM
109	-	116750	116896	144	48					
110	-	116971	117372	399	133					TM
111	+	117179	117469	288	96					
112	+	117529	118617	1086	362					TM
113	+	118674	118928	252	84	sortilin-related receptor precursor	Acyrthosiphon pisum	42	9 × 10^-9^	LDLRA_2
114	+	118723	118911	186	62					TM
115	+	119035	119493	456	152					TM

PmNV094 showed 28% identity with helicase from HzNV-1 and HzNV-2 (BLASTP: E = 3 × 10^-19^; Table 4) and was predicted to contain the primase_Cte domain (CDD: E = 4.99 × 10^-7^). In the phylogenetic tree based on the helicase protein sequences of baculoviruses and nudiviruses, which also had a high bootstrap support percentage at each node, PmNV again formed a cluster with HzNV-1 and HzNV-2 (Figure 2B). As with *dnapol*, evolutionary distances between the nudiviruses and PmNV are still greater than those within the baculoviruses.

#### RNA polymerase subunits and an RNA transcription initiation factor

Five putative genes, PmNV014 (*p47*), PmNV023 (*lef-8*), PmNV052 (*lef-5*), PmNV058 (*lef-9*) and PmNV091 (*lef-4*), are homologous to those involved in baculovirus transcription. These genes are expressed in the late or very late infection stage, and they play different transcriptional roles. LEF-5 is a transcription initiation factor [21], while P47, LEF-4, LEF-8 and LEF-9 are RNA polymerase subunits. Although *p47* and *lef-9* are fused into a single gene in HzNV-1 and HzNV-2, all five of the genes in the transcriptional group are conserved between the baculoviruses and nudiviruses (Table 2), which suggests that the baculoviruses and nudiviruses share a similar mode of transcription.For the phylogenetic analysis (Figure 2C), the protein sequences of LEF-4, LEF-5, LEF-8, LEF-9 and P47 from the nudiviruses and selected baculoviruses were arranged sequentially and then aligned. In this tree, PmNV also formed a cluster with the nudiviruses. The tree showed a relatively large evolutionary distance between the baculovirus cluster and the nudivirus cluster.

#### Genes involved in oral infectivity

PmNV010 (*pif-5/odv-e56*), PmNV015 (*pif-2*), PmNV039 (*pif-1*), PmNV072 (*p74*), PmNV088 (*pif-6*), PmNV093 (*pif-3*) and PmNV096 (*pif-4*) were homologous to genes involved in *per os* infection. These seven genes are conserved across the baculoviruses and nudiviruses, and they are all essential for the occlusion derived virus (ODV) infectivity of the baculoviruses, either by specific binding to the midgut cells (P74, PIF-1 and PIF-2) [22, 23] or by a mechanism that has not yet been determined (PIF-4, PIF-5 and PIF-6) [24–26]. Although PIF-3 was shown not to be involved in specific attachment and fusion [23], deletion of its N-terminal nuclear localization signal prevents *per os* infectivity [27].

The shared importance of these *per os* infectivity factors implies that despite the absence of OBs in the nudiviruses, these viruses nevertheless all share a very similar oral infection mechanism. Moreover, four homologous genes that have relatively high identity and similarity (*p74, pif-1, pif-2 and pif-3*) are also found in the hytrosaviruses, indicating that the baculoviruses, nudiviruses and hytrosaviruses all have the same important ancestral conserved model for virus infectivity. Interestingly, BLASTP search showed that PmNV015 (*pif-2*) most closely matched *Macrobrachium* nudivirus (MRNuV) PIF-2 (Accession No. : AFP33714) with an identity of 66%. To date, only one other MRNuV gene has been sequenced (IAP), but we note that unlike the other insect nudiviruses, both PmNV and MRNuV are aquatic viruses, and it is possible that these two viruses - perhaps together with *Penaeus vannamei* single nucleopolyhedrovirus, PvSNPV - are quite closely related.

The constructed phylogenic tree of the oral infectivity genes *p74*, *pif-1*, *pif-2* and *pif-3* (Figure 2D) is similar to the DNA polymerase and Helicase trees, providing further evidence that PmNV is more closely related to the nudiviruses than the baculoviruses.

#### Proteins related to viral packaging, assembly, and release

There are 18 baculovirus core genes involved in viral structure formation, but only four of these genes have homologs that could be found in the PmNV genome: *p33* (PmNV008), *vp91* (PmNV009), *vlf-1* (PmNV056 and PmNV090) and *38 k* (PmNV059) (Table 2). The PmNV genome even lacks a homolog to *vp39*, which is found in the nudiviruses and is thought to encode the major capsid protein [28].

P33 is a component of the occluded virions of AcMNPV, CuniNPV (*Culex nigripalpus* nucleopolyhedrovirus) and HzSNPV (*Helicoverpa zea* single-nucleocapsid nucleopolyhedrovirus) [29–31]. AcMNPV P33 has been shown to interact with the human tumor suppressor p53 to enhance p53-induced apoptosis [32]. However, while AcMNPV P33 also interacts with *Spodoptera frugiperda* P53 (SfP53) and oxidizes SfP53 *in vitro*, it does not enhance SfP53-mediated apoptosis in Sf9 cells [33]. AcMNPV P33 has also been demonstrated to have sulfhydryl oxidase activity [34] and it is required for budded virus production and for the formation of multiply enveloped occlusion-derived nucleocapsids [35]. VP91 is expressed at the late stage of viral infection and is present in the capsid structure of OpMNPV (*Orgyia pseudotsugata* multicapsid nucleopolyhedrovirus) [36]. VLF-1 is expressed at the very late stage and is involved in the production of the AcMNPV nucleocapsid [37]. Gene 38 K is essential for budded virus formation and the nucleocapsid assembly of AcMNPV [38]. Taken together, the low number of homologs in this functional group indicates that the baculoviruses and nudiviruses are highly divergent in terms of their viral structural proteins.

#### Proteins of unknown function

The best match to PmNV086 was the Ac81-like protein of HzNV-1 and HzNV-2 (BLASTP: E = 3 × 10^-21^) (Table 4). In BmNPV (*Bombyx mori nucleopolyhedrovirus*), the Ac81 homolog (ORF67) is a non-structural protein of unknown function that interacts with host actin [39]. The protein sequence encoded by PmNV086 was predicted to have a transmembrane domain by SMART, and Pfam found a partial match (E = 0.16) in the eukaryotic RHD3 protein (Root hair defective 3 GTP-binding protein).

#### Non-core AcMNPV genes

We found four other putative PmNV ORFs homologous to baculovirus genes that are not included in the 37 core genes: PmNV006 (*methyltransferase*), PmNV034 (*odv-e66*), PmNV036 (*odv-e66*), PmNV106 (*iap*) (Table 2).

Methyltransferase is expressed in the late phase of infection and has cap O-dependent methytransferase activity [40]. However, deletion studies with methyltransferase-defective AcMNPV have shown that it is not essential for the budded virus or for occluded virus production, and that it has no effect on AcMNPV [41].

The PmNV genome has two neighboring ORFs with the same orientation (PmNV034 and PmNV036) that are homologous to *odv-e66.* ODV-E66 is a late expression, structural component of occluded-derived virus (ODV) envelopes [42]. The N-terminus of ODV-E66 contains a highly hydrophobic INM-sorting (inner nuclear membrane-sorting) motif that traffics AcMNPV viral proteins to enhance viral assembly [43]. However, despite their homology, PmNV034 and PmNV036 have different lengths, relatively low identity and similarity (38% and 53%, respectively), and the hydrophobic INM-sorting sequence was found only in PmNV036 and not in PmNV034. AcMNPV ODV-E66 has also recently been shown to act as a chondroitin lyase [44]. Interestingly, both PmNV034 and PmNV036 contain all five of the conserved catalytic residues that are found in AcMNPV ODV-E66, suggesting that either or both of these proteins might function as a chondroitin lyase.

PmNV106 contains a single predicted BIR (baculoviral inhibitor of apoptosis repeat) domain. This domain acts directly on the caspase family of protease enzymes to block apoptosis and thereby allow the virus more time to replicate. The IAP (inhibitor of apoptosis) family of proteins contains 1 ~ 3 BIR domains in the N terminal and an optional RING domain in the C terminal. PmNV106, which lacks a predicted RING domain and is shorter than most other IAPs (Additional file 8: Table S6 and Additional file 9: Figure S3), shares an identity of less than 60% with other IAPs.

The PmNV genome contains two ORFs with some homology to polyhedrin, PmNV001 and PmNV020. LC-MS analysis has shown that the polyhedrin protein purified from PmNV occlusion bodies is derived from PmNV001 [19]. Meanwhile, although three nudivirus ORFs (Hz1V068, OrNV ORF16 and GbNV ORF65) were previously reported to show some homology to AcMNPV polyhedrin [18], and our BLASTP search also found a fourth match in Hz2V070, there is no functional evidence that polyhedrin protein is actually expressed by any of these ORFs. The evolutionary relationship between these low identity, possible polyhedrin nudivirus homologs and baculoviral polyhedrin still remains unclear, but we tentatively conclude that PmNV020 is unlikely to be a polyhedrin gene.

PmNV polyhedrin is discussed in more detail in the second half of this study.

### PmNV putative ORFs that are homologous to nudivirus, bracovirus and hytrosavirus genes

#### Enzymes involved in DNA replication

The PmNV genome contains four putative ORFs, PmNV 020 (*DNA excision and repair*) PmNV055 (*integrase*), PmNV076 (*helicase2*) and PmNV079 (*helicase2*), that are homologous to genes involved in DNA replication.

PmNV020, in addition to being a homolog to four nudivirus ORFs that are homologous to AcMNPV polyhedrin, has a predicted 29% amino acid identity with Hz1V068 and Hz2V070 (BLASTP: E = 3 × 10^-14^). A Pfam database search revealed that the N-terminus of all three proteins matched the XPG (Xeroderma pigmentosum complementation group G) N-terminal domain with E-values of around 0.03 (PmNV020 33-110 aa = 0.03, Hz1V068 = 0.027 and Hz2V070 = 0.028). The presence of this domain in Hz2V070 led Burand *et al*. [7] to propose that it is a DNA excision and repair enzyme that restores damaged DNA. Homologs of this enzyme are found among the nudiviruses and in the *Musca domestica* salivary gland hypertrophy virus (MdSGHV), but not in the baculovirus genomes. The baculoviruses encode various other proteins for DNA repair instead. For example, some of the nucleopolyhedroviruses use photolyase to repair UV-damaged DNA [45]. PmNV055 (*integrase*) most closely matched the integrase of HzNV-1 (BLASTP: E = 1 × 10^-32^) (Table 4), which phylogenetic analysis suggests was formed by duplication of the baculovirus and nudivirus conserved *vlf-1* gene [46]. PmNV055 is predicted to belong to the phage integrase family (Pfam: PmNV055 105-278 aa, E = 7.8 × 10^-16^). Members of this family mediate unidirectional site-specific recombination between two DNA recognition sequences [47]. Tyrosine is the key catalytic site of this enzyme activity, and a catalytic tyrosine was found on one of the six possible active sites of PmNV055 predicted by Pfam. Integrase mediates the excision and integration of viral genetic material into the host genome. This process is essential for *Microplitis demolitor* bracovirus (MdBV) [48], and HzNV-1 also has a similar mechanism that integrates viral DNA into the host chromosome for persistent infection [49]. However, it is still unclear if this protein has any functional role in PmNV.

BLASTP found that PmNV076 and PmNV079 both matched the same *Danaus plexippus* protein with an identity of 30% (E = 1 × 10^-46^) and 28% (E = 5 × 10^-39^), respectively (Table 4). The same two ORFs also matched HzNV-1 putative helicase 2 with 27% identity (BLASTP: E = 1 × 10^-39^ and 7 × 10^-35^, respectively). However, these two ORFs have relatively low amino acid sequence identity and similarity (17% and 35%, respectively), which means that if they really are homologs to helicase 2, then the original gene duplication must have occurred a long time ago. The genomes of the other sequenced nudiviruses each contain only a single instance of a gene that is homologous to helicase 2.

#### Structural proteins

Four putative ORFs, PmNV045 (*PmV-like*), PmNV069 (*HzNVORF106-like*), PmNV090 (*HzNVORF140-like*) and PmNV107 (*HzNVORF9-like*), were homologous to bracovirus proteins. Proteomic analysis showed that these proteins were associated with the bracovirus particles [50], suggesting that the PmNV proteins might also be structural proteins involved in viral packaging, assembly, or release.

#### Proteins with other known and unknown functions

Fourteen other PmNV ORFs are homologous to nudivirus and bracovirus genes/ORFs with miscellaneous or unknown functions.

PmNV024 most closely matched the HzNV-1 *p51* gene (BLASTP: E = 4 × 10^-9^) (Table 4), which is a late expression gene that putatively acts as a late regulator with unknown function [51].

PmNV038 and PmNV046 have both been annotated previously as *thymidine kinase* (*tk*) [4]. However, we found that PmNV038 was homologous to Hz2V034, which has a guanosine monophosphate kinase (GMPK) motif at position 57-96 aa and is annotated as a guanosine monophosphate kinase [7]. In Hz1V111, Hz2V034 and PmNV038, the conserved catalytic GMPK motif, G-X_2_-G-X-G-K has one mismatched amino acid at the second G position, but it is not known if this affects the putative functionality of these proteins. PmNV046 contains an AAA (ATPases associated with diverse cellular activities) domain (Pfam: E = 0.0013), which implies that this protein belongs to the large, functionally diverse P-loop NTPase family. Proteins in this family are involved in a range of biological processes, including DNA replication, gene regulation, protein degradation, and signal transduction.

PmNV098 most closely matched HzNV-2 esterase/lipase (BLASTP: 3 × 10^-4^) (Table 4). These proteins include the Aes (prokaryotic acetylesterase) signature (CDD: E = 6.19 × 10^-5^), which suggests that PmNV098 might function as an enzyme involved in lipid metabolism.

Most of the PmNV homologs were found in the genomes of HzNV-1 and HzNV-2, and there was a higher similarity to genes from these two species than to the genes from other species. This is consistent with the results of the phylogenetic analysis, and provides further evidence that among the completely sequenced viruses, the *Heliothis zea* and *Helicoverpa zea* nudiviruses are the two species most closely related to PmNV.

### Occlusion bodies of PmNV

**Figure 3:**
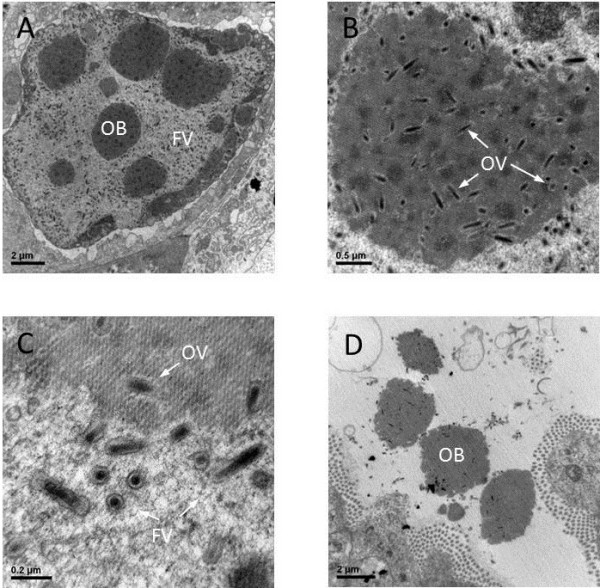
**Transmission electron micrographs of PmNV-infected postlarval** ***Penaeus monodon*** **hepatopancreatic cells. (A)** Occlusion bodies (OBs) and free virions (FV) in the nucleus. **(B)** Occluded virus (OV) in the occlusion body which consists of a polyhedrin matrix. **(C)** Free virions in the nucleus. **(D)** Mature released OBs in the tubule lumen of the hepatopancreas.

Although the results of our phylogenetic analysis and search for ORF homologs suggest a close relationship between PmNV and the non-occluded nudiviruses, Figure 3 shows that a PmNV -infected nucleus contains enveloped virions that are embedded in approximately spherical occlusion bodies (OBs). The production of these OBs is one of the main reasons why PmNV was previously assigned to the baculoviruses.

An OB is a highly ordered crystalline protein matrix produced by some insect viruses (*Baculoviridae*, *Entomopoxvirinae* and *Cypovirus*) and shrimp viruses (PmNV and PvSNPV) that enables the virus to survive in a hostile environment. OBs protect the virions for long periods, and in baculoviruses they act as a *per os* delivery system by resisting solubilization until exposed to the host insect’s alkaline midgut [52]. The OBs of PmNV consist of a polyhedrin protein that does not have any significant sequence similarity to any known proteins in GenBank, but TBLASTN found a partial PvSNPV nucleotide sequence of unknown function that had 47% identity and an E-value 5 × 10^-72^ (Accession No. : DQ496179) [19]. It has not yet been shown whether or not this sequence is involved in the formation of PvSNPV OBs. However, both PmNV and PvSNPV have distinct, unenveloped spherical and tetrahedral OBs, respectively, and these are quite different from the enveloped OBs of the insect baculoviruses [53].

### Recombinant PmNV polyhedrin spontaneously assembles into a protein complex

**Figure 4:**
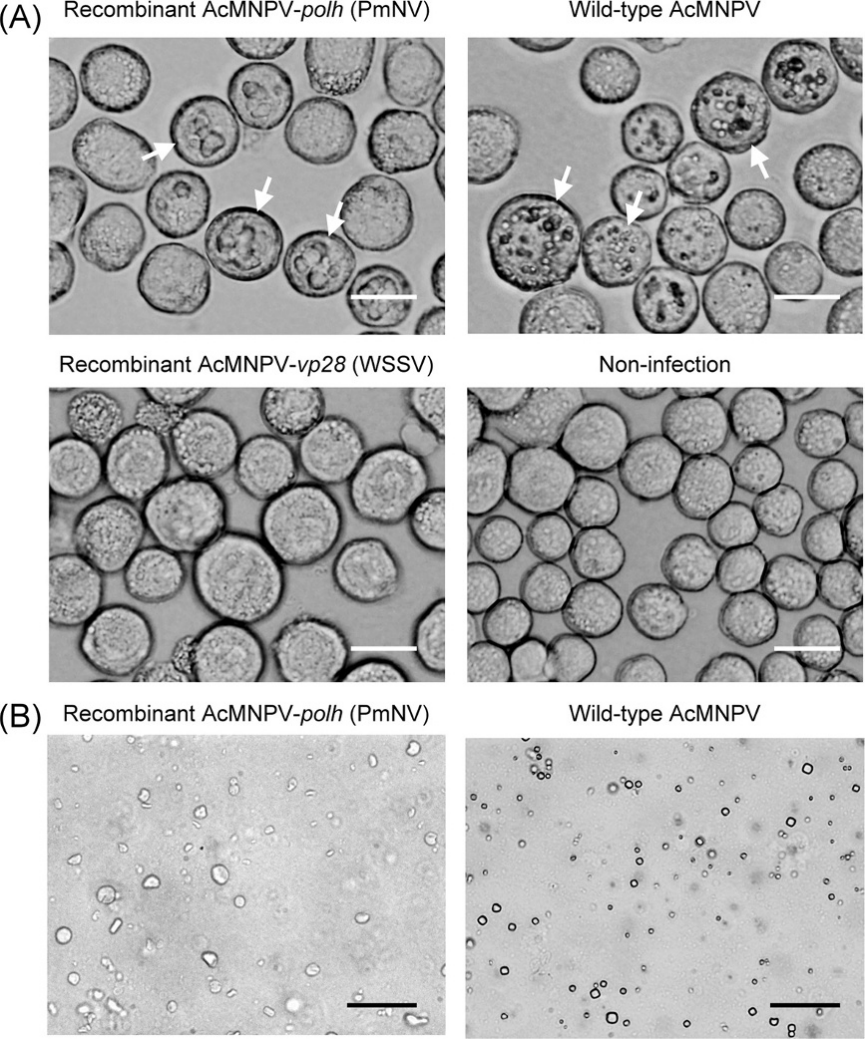
**The polyhedrin protein matrix and occlusion bodies (OBs) production of recombinant AcMNPV-** ***polh*** **(PmNV) and wild-type AcMNPV in Sf9 at 3 days post-infection. (A)** Photomicrographs of Sf9 cells infected with recombinant AcMNPV-*polh* (PmNV), wild-type AcMNPV, recombinant AcMNPV-*vp28* (WSSV) as non-occluded control and non-infected cells (Normal cells). Examples of cells producing polyhedrin protein matrix and OBs are indicated by arrows. **(B)** Photomicrographs of purified polyhedrin protein matrix and AcMNPV OBs. Scale bars are 20 μm.

To study the molecular characteristics of PmNV polyhedrin, we expressed the PmNV *polyhedrin* gene (PmNV001) using an AcMNPV baculovirus expression system. Sf9 cells were then infected with recombinant AcMNPV-*polh* (PmNV) (i.e., AcMNPV with its normal polyhedrin gene replaced by that of PmNV) or wild-type AcMNPV (i.e., carrying its normal polyhedrin gene) and by 3 dpi, significant numbers of occlusion bodies had accumulated in the nuclei of both infected cell types (Figure 4A). By contrast, cells infected with recombinant AcMNPV-*vp28* (WSSV) were swollen and had a larger nucleus than the normal uninfected cells, but no occluded protein packages were seen inside the cells (Figure 4A). This result indicates that PmNV polyhedrin can aggregate into a protein matrix naturally and form a stable structure in the cells. After 5-7 days of virus infection, most of the cells showed a serious cytopathic effect, and yielded large amounts of polyhedrin protein and AcMNPV OBs.After purification of the recombinant PmNV polyhedrin matrices and the AcMNPV OBs, the light-refractive polyhedral AcMNPV OBs were more easily visualized than the irregularly shaped and larger recombinant PmNV polyhedra (Figure 4B). This result suggests that the assembly of PmNV polyhedrin might need other viral or host factors that are absent from the insect expression system to form PmNV wild type spherical OBs.

### The effect of DAS buffer on the PmNV polyhedrin protein matrix and the AcMNPV OBs

**Figure 5:**
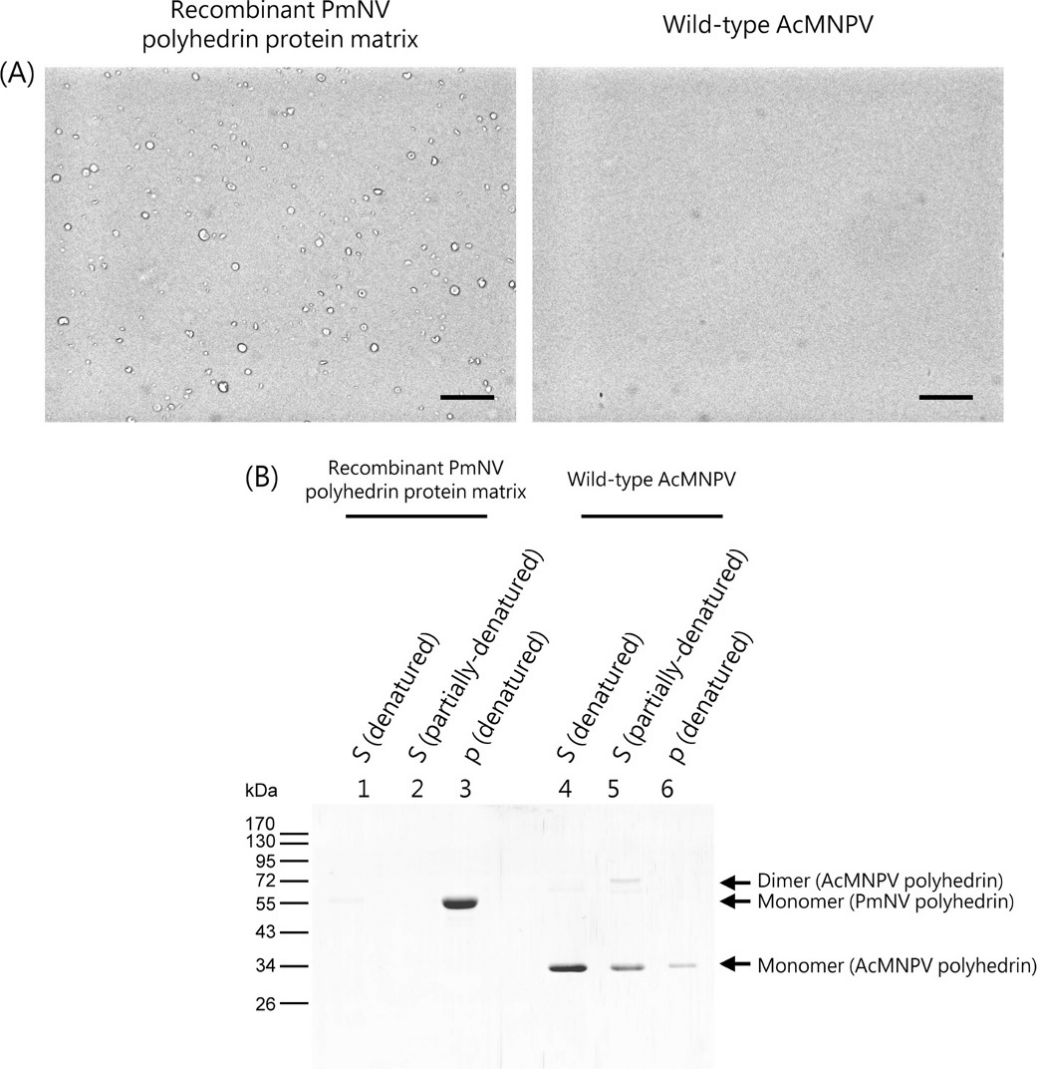
**Effect of DAS buffer on purified recombinant polyhedrin matrix and AcMNPV OBs. (A)** Photomicrographs of resuspended pellets after centrifugation showing intact PmNV polyhedrin matrix but no AcMNPV OBs. Scale bar represents 20 μm. **(B)** SDS-PAGE analysis of the denatured and partially denatured supernatant and the denatured pellet. The results show that DAS buffer did not solubilize the recombinant PmNV polyhedrin (S lanes 1 & 2) but did solubilize polyhedrin from AcMNPV OBs (S lanes 4 & 5). S: supernatant; P: pellet.

Dilute alkaline saline (DAS) buffer is commonly used for enhancing *in vitro* dissolution of baculoviral OBs, and as expected, the polyhedrin from the AcMNPV OBs was found to be entirely dissolved in the supernatant fractions (Figure 5). However, the recombinant PmNV polyhedrin was not dissolved and was found in the pellet. We also note that the AcMNPV polyhedrin bands show that both monomers and dimers were present in the partially denatured fraction.

### Recombinant PmNV polyhedrin is solubilized by 2% SDS, 8 M urea and acid

**Figure 6:**
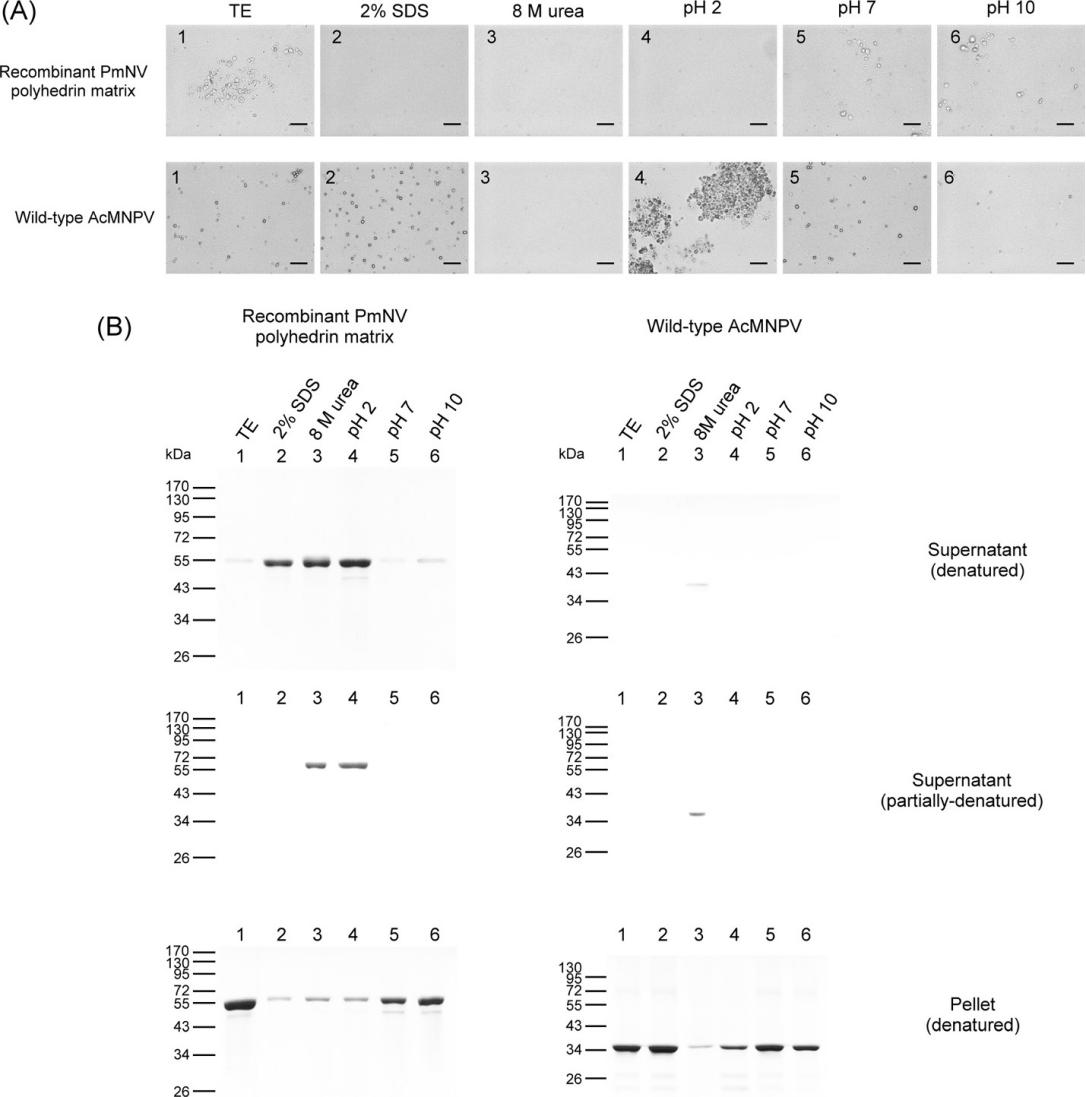
**Solubility of purified recombinant PmNV polyhedrin and wild-type AcMNPV. (A)** Photomicrographs of pellet samples after indicated treatments showing presence or absence of PmNV polyhedrin matrix or AcMNPV OBs. Scale bars 50 μm. **(B)** SDS-PAGE analysis of pellets and supernatant solutions from treatments in **(A)**. Lane 1: TE buffer (negative control); Lane 2: 2% SDS; Lane 3: 8 M urea; Lane 4, 5 and 6: phosphate buffer adjusted to pH 2, pH 7 and pH 10, respectively.

Additional solubility analysis revealed that 2% SDS and 8 M urea were able to dissolve recombinant PmNV polyhedrin by respectively breaking the protein’s hydrophobic interactions and hydrogen bonding (Figure 6). Acid treatment was also able to dissolve the PmNV polyhedrin. We further note that when the supernatant fraction was only partially denatured, the presence of the polyhedrin bands after 8 M urea and acid treatment shows that only these two treatments were able to induce complete dissolution of the PmNV polyhedrin matrix to the monomeric protein.

By contrast, when the same treatments were applied to purified AcMNPV OBs, only 8 M urea was able to induce dissolution of the OBs into polyhedrin monomers and an extremely faint band of dimers (Figure 6B). Although most of the AcMNPV polyhedrin was solubilized in the presence of 8 M urea, the bands in lane 3 of the supernatant fractions were fainter than expected. We speculate that this was because the solubilized polyhedrin was digested by a protease that became active in the presence of urea. We also note that although the AcMNPV OBs were soluble in DAS (Figure 5), alkaline conditions alone (pH 10) were not sufficient to dissolve the OBs. This difference may be due to the presence of sodium, carbonate, and EDTA in the DAS buffer [54].

### Wild-type PmNV OBs

After purifying wild-type PmNV OBs from the hepatopancreas of PmNV-infected postlarvae, we found that, unlike baculoviral OBs, the PmNV OBs were not spun down by 4,000 × *g* centrifugation for 10 min (Additional file 10: Figure S4). Finally, however, we found that 13,000 × *g* centrifugation for 30 min was sufficient to spin down most of the OBs, although some OBs still remained in the supernatant.

**Figure 7:**
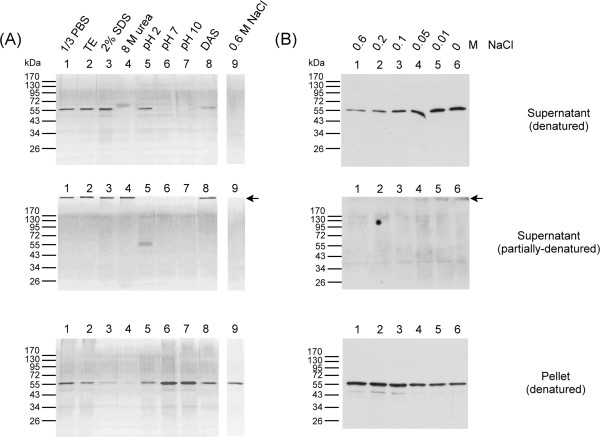
**Solubility of purified wild-type PmNV OBs in various buffers and different concentrations of NaCl. (A)** SDS-PAGE of supernatant solutions (soluble) and pellets (insoluble) after PmNV OBs were subjected to the treatments indicated. Black arrows indicate partially denatured PmNV OBs. **(B)** Western blots of supernatant solutions (soluble) and pellets (insoluble) from PmNV OBs treated with various concentrations of NaCl and showing increasing solubility as salinity decreases.

A further examination of the effects of different buffers on the PmNV OBs found that only acidic conditions were able to induce PmNV polyhedrin to dissociate into monomers under partially denatured conditions (Figure 7). This result is consistent with the above analysis of the recombinant PmNV polyhedrin protein matrix, and also suggests a possible PmNV transmission mechanism whereby its OBs are dissociated in the acidic environment of the host’s digestive tract. Even though both insect and PmNV OBs are transmitted *per os*, they show different characteristics that appear to be specifically adapted to the digestive environment of their respective hosts.We also found that wild-type PmNV OBs were solubilized in 1/3 PBS, TE, 2% SDS, 8 M urea and DAS buffer, but were stable in neutral and alkaline phosphate buffer (pH 7 and pH 10) and in 0.6 M NaCl (close to the salt concentration of seawater) (Figure 7A). (The identity of the slight shifted band in lane 4 of the denatured supernatant fraction was confirmed by Western blotting [data not shown]; the shift itself was probably caused by the high concentration of urea.) A high molecular weight polyhedrin complex was observed in the supernatant under partially denatured conditions (Figure 7A, black arrow). The identity of the polyhedrin in these high molecular weight bands was confirmed by LC-MS/MS analysis (data not shown). The results shown in Figure 7A further suggest that ionic strength might affect the solubility of the PmNV OBs, specifically that at higher ionic strengths, the PmNV OBs are more likely to be present in the pellet fraction. To better understand this relationship, we examined the effect of different salt concentrations on the PmNV OBs, and found that solubility increased from 0.6 M NaCl to 0 M NaCl (Figure 7B).

In addition to showing the basic features of PmNV OBs, these results suggest that instead of the more typical protective role of the AcMNPV OBs, PmNV might in fact use OBs as part of its natural transmission strategy. One possible pathway of viral transmission is as follows: After the mature PmNV OBs are released, the high ionic strength of the seawater environment helps to maintain the integrity of the PmNV OBs and even allows physical association of large numbers of OBs with shrimp feces or the bodies of dead shrimp. Subsequently, when healthy shrimps ingest these PmNV OBs, the OBs become solubilized in the lower ionic strength conditions of the shrimp’s digestive tract, and then dissociate in the acid midgut. After the virions are released from the OBs, they can then infect the midgut epithelium cells to complete the viral transmission.

## Conclusion

**Figure 8:**
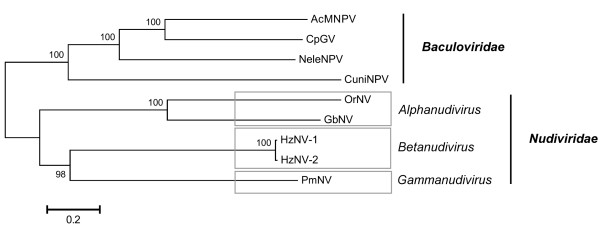
**Phylogenetic analysis of the concatenated protein sequences of LEF-4, LEF-5, DNA polymerase and Ac81 from PmNV, the nudiviruses, and four baculoviruses.** This analysis follows Jehle *et al.*[5]. The tree was inferred using MEGA 5.2 and the neighbor-joining method. Robustness was tested using bootstrap (1000) analysis. Percent values are indicated at the nodes. Jehle *et al*. [5] proposed that the nudiviruses should be assigned to one of two new genera. *Alphanudivirus* and *Betanudivirus*. We propose here that PmNV should be assigned to a third new genus, *Gammanudivirus*.

Nudiviruses were historically regarded as baculoviruses because both groups share similar characteristics, including rod-shaped, enveloped virions, a circular dsDNA genome, and replication in the infected nucleus. Eventually, in 1995, nudiviruses were removed from the family *Baculoviridae* due to low genetic similarity [55], but their taxonomic classification continues to be debated. While PmNV has characteristics that are shared both by the nudiviruses and the baculoviruses, it initially seemed more like a baculovirus than a nudivirus because of its OBs. However, the results of the present study provide further evidence that this relatively unique virus does not comfortably belong to any of the present baculovirus genera. Instead, we would argue that PmNV properly belongs within the newly proposed taxonomy of the *Nudiviridae* family [5]. This is despite the fact that our phylogenetic analysis suggests that the evolutionary distance between PmNV and the nudiviruses is greater than the distances between any of the four genera (*Alphabaculovirus, Betabaculovirus, Gammabaculovirus* and *Deltabaculovirus*) of the *Baculoviridae* (Figure 2). We also recognize that the host range of PmNV is distinct from that of the other nudiviruses. Nevertheless, when we followed Jehle *et al.* [5] and used several taxonomically important genes to construct a phylogenetic tree, we found that, as in Figure 2, PmNV once again clearly forms a separate clade within the nudiviruses (Figure 8). We therefore propose that PmNV should be assigned to a third nudivirus genus, *Gammanudiviruses*.

## Methods

### Virus purification

The PmNV used in this study was originally collected from infected *Peneus monodon* Indonesia on May 23, 2007, and provided to us by the GeneReach Biotech biotechnology company (stored at -80°C). To prepare purified virus, *P. monodon* postlarvae (PL2) were infected *per os* with PmNV by feeding them with infected postlarvae that had died. Infection was monitored by randomly collecting PLs, and using a microscope to examine the PmNV occlusion bodies in squash mounts of hepatopancreas stained with 0.05% malachite green. In addition, PmNV infection status was confirmed using an IQ2000 detection kit (GeneReach). At 2 weeks after infection, severely infected PL15 were collected and stored at -80°C. Frozen PmNV-infected PL15 were homogenized in TESP buffer (50 mM Tris-HCl, 500 mM NaCl, 10 mM EDTA and 200 mM PMSF, pH 8.5) using a glass pestle (kept on ice), with the addition of TESP with 10% sucrose buffer (final volume 50 ml). To remove the occlusion bodies and cell debris, this mixture was then centrifuged (8,000 × g for 20 min at 4°C) and the supernatant was filtered successively using gauze, followed by 0.8 and 0.45 μm filters. Thereafter, the free extracellular virions in the filtered supernatant were centrifuged (30,000 × g for 1 h at 4°C). The supernatant was removed, TESP with 10% sucrose buffer was added and the mixture was centrifuged (30,000 × g for 1 h at 4°C) to wash the pellet. The supernatant was discarded and the pelleted virus was re-suspended in TM buffer (50 mM Tris-HCl, 10 mM MgCl_2_, pH 7.5).

### Genomic DNA preparation

Two methods were used to obtain viral genomic DNA. As explained below, the first method used DNase I treatment and gel elution to achieve higher purity, while the second method did not use DNase I.

#### DNase I treatment of PmNV virions and viral genomic DNA purification

Purified virus was treated with 0.25 μg/μl DNase I (Bioshop) at room temperature for 1-2 h to digest the host DNA. Then, virions were collected by high speed centrifugation (30,000 × *g* for 1 h at 4°C). Viral genomic DNA was extracted by the DTAB/CTAB method (GeneReach), and then separated on a 0.6% agarose gel; the DNA comprised a high molecular weight major band, which was transferred to DE-81 paper (Whatman) by electrophoresis. This paper (with the DNA) was placed into a custom-made plastic spin column and washed twice with 200 μl low salt buffer (10 mM Tris-HCL pH 7.5, 1 mM EDTA, and 100 mM LiCl) by centrifugation (30,000 × g for 1 h at 4°C). Thereafter, DNA was eluted with 200 μl of high salt buffer (10 mM Tris-HCl pH 7.5, 1 mM EDTA and 1 M LiCl) and 200 μl n-butanol was added to remove EtBr. The genomic DNA was then precipitated overnight at -80°C by adding 20 μl 3 M NaOAc (pH 5.2) and 550 μl 100% ethanol. After centrifugation, the supernatant was discarded, and the DNA pellet was washed with 200 μl 70% ethanol and air-dried for 5-10 minutes. Finally, the DNA was dissolved in 10 μl ddH_2_O and stored at -20°C.

#### Non-DNase I treatment of PmNV

For this step, DNA was extracted using DTAB/CTAB method (GeneReach) described above, with the exception of DNaseI treatment.

### Genome sequencing

Viral genomic DNA that had been treated with DNase I was initially used for high-throughput sequencing. First, about 50 ng of this DNA was used for a fragment library (insert length: 300-500 bp) using the Chip-seq method to amplify DNA (which increased PCR bias) and a paired-end method was used for sequencing following the Ovation® Ultralow Library System instruction. Subsequently, viral genomic DNA not treated with DNase I (DNA concentration is about 10 μg) was used for the second high-throughput sequencing, preparing the DNA library according to Miseq instruction (average insert length: 170 bp). The Cutadapt program (http://code.google.com/p/cutadapt/) was used to trim the raw data and to reject poor-quality reads. CLC Genomics Workbench 5.1 software (CLC bio) was used for *de novo* sequence assembly. Based on the longest contig, 240 primers were designed to amplify ~1,500 nt PCR fragments with 500 nt overlaps (see Additional file 1: Table S1 in the supplemental material for primer sequences). These fragments, which covered the entire virus genome, were sequenced by the Sanger method (first generation sequencing). Subsequently, 134 additional primers (Additional file 1: Table S1) were designed to verify sequences with unreliable signals.

### DNA sequence analysis

Repetitive sequences were identified by tandem repeat finder [56] and DNAMAN software (Version 7, Lynnon Corporation, Pointe-Claire, QC, Canada). The ORF predictions were performed by DNAMAN (parameter: coding more than 50 amino acid), GLIMMER3 [57] (parameter: standard code and circular genome type), GeneMarkS [58] (parameter: Intronless eukaryotic-virus) and FgenesV0 (standard code and circular genome type; http://linux1.softberry.com/berry.phtml) programs. Any ORF predicted by at least two programs or with an E-value less than 1 of BLASTP (http://blast.ncbi.nlm.nih.gov/) were subjected to a database search to identify possible functions. The protein sequence of predicted ORFs were further searched for conserved domains and structure prediction by PROSITE [59], SMART [60], Pfam [61] and HHpred [62] by the online database. Protein sequence comparisons (Tables 2 and 3) were aligned by ClustalX2 [63] and protein identity and similarity were characterized using GeneDoc [64].

### Transmission electron microscopy

Infected hepatopancreas (4-8 d post-infection) were prefixed in 2.5% glutaraldehyde and 2% paraformaldehyde in 0.1 M phosphate buffer (PBS) at 4°C overnight. Samples were transferred to a glass tube and rinsed with 0.1 M PBS (3 times, each for 10 min). Then, 1% osmium tetroxide was used to postfix the tissue at room temperature for 90 min. Fixed samples were treated with 0.2% uranyl acetate (overnight at 4°C) and dehydrated on ice (serial concentrations of 70, 80, 90 and 95% alcohol, each for 10 min). The final step of dehydration used two exposures to absolute alcohol at room temperature (each 15 min), followed by two exposures to acetone (each 15 min). Samples embedded in Spurr-Epon were cut into ultrathin sections on a Reichert OMU ultramicrotome and stained with uranyl acetate and lead citrate.

### Expression and purification of PmNV polyhedrin matrix

The PmNV *polyhedrin* gene (PmNV 001) was cloned into pBacPAK9 (Clontech) (an expression system based on the baculovirus AcMNPV genome in which the original viral polyhedrin gene has been replaced by a cloning site for heterologous gene expression) with *Bam*HI and *Not*I restriction sites (p5841-polhFL-BamHI-F: 5′-GCCGGATCCATGTTCGACGATAACATGATG-3′ and p5842-polhFL-NotI-R: 5′-GATTGCGGCCGCTTCATTTGTATGATGCGTCTT-3′) and recombinant baculoviruses were produced by co-transfection of linearized baculoviral DNA, using BacPAK™ Baculovirus Expression System (Clontech) in accordance with manufacturer’s instruction. Then, Sf9 cells (Novagen) were cultured in SF900™ II SFM (Gibco) medium supplemented with 10% FBS (Gibco) at 28°C. In Sf9 cells infected with P2 progeny of PmNV polyhedrin recombinant baculoviruses, a polyhedrin protein matrix was present. At 7 d after infection, Sf9 cells with overt signs of infection were lysed (TE buffer with 0.1% SDS, vortexed for 5 min), washed twice with TE buffer, and recombinant polyhedrin protein matrices collected by centrifugation (4,000 × *g* for 10 min).

Wild-type AcMNPV strain (AcMNPV-TWN4), isolated from beet armyworm, *Spodoptera exigua* [65, 66], was provided by Professor Chung-Hsiung Wang (National Taiwan University). At 7 d after AcMNPV infection, Sf9 cells were lysed by TE buffer with 2% SDS and sonicated for 5 min. The AcMNPV were obtained by centrifugation (4,000 × *g* for 10 min) and washed twice by TE buffer.

The WSSV (White spot syndrome virus) *vp28* gene was cloned into pBAC-1 transfer plasmid (Novagen) with *Eco*RI and *Xho*I restriction sites (p5274-VP28-EcoRI-F: 5′-GCCGAATTCATGGATCTTTCTTTCACTCTTTCGGTC-3′ and p5275-VP28-XhoI-R: 5′-GTGCTCGAGCTCGGTCTCAGTGCCAGAGTAGGTGAC-3′) and recombinant baculoviruses were produced by co-transfection of baculoviral DNA by BacVector®-1000 transfection kit (Novagen), following the manufacturer’s instructions.

### Purification of the wild-type PmNV OBs

Infected hepatopancreases were homogenized by plastic pestle in 200 μl of 1/3 PBS. The cell debris was pelleted and removed by centrifugation (4,000 × *g* for 10 min). The PmNV OBs were in the supernatant fraction and were subsequently pelleted by centrifugation (13,000 × *g* for 30 min).

### Dissolution assays of recombinant polyhedrin protein matrix, AcMNPV OBs and PmNV OBs

Purified polyhedrin protein matrix and AcMNPV OBs or PmNV OBs were aliquoted into each tube and centrifuged (4000 × *g* for 10 min or 13000 × *g* for 30 min, respectively). The supernatant was removed and then each buffer (DAS buffer, 1/3 PBS, TE buffer, 2% SDS, 8 M urea in 20 mM Tris, 3 phosphate buffer with different pH value (phosphoric acid were adjusted to pH 2, pH 7 and pH 10 by NaOH and HCl) or 0.6 M NaCl) was added to the pellet in each aliquot for 5 min with vortexing. After incubation and centrifugation, the supernatant and pellet were separately collected and subjected to SDS-PAGE. Supernatant fractions were denatured by mixing 10 μl of supernatant with 10 μl of 2 × Laemmli buffer [67] and boiling the mixture. Partially denatured supernatant fractions were prepared by mixing 10 μl of supernatant with 10 μl of 2 × Laemmli buffer without β-mercaptoethanol and boiling the mixture. Denatured pellet fractions were prepared by adding 20 μl of 1 × Laemmli buffer and boiling the mixture. Thereafter, proteins were separated using SDS-PAGE.

### Polyclonal antibody production and Western blotting

The C-terminus of polyhedrin (residues 251-452) [19] was amplified and sub-cloned to pET-16(b) expression vector (Novagen) with *Nde*I and *Bam*HI restriction sites (p5786-polh-251-NdeI-F: 5′-CGTCATATGTCAGAAAATACTTCTATACAA-3′, p5787-polh-452-BamHI-R: 5′-GCCGGATCCTTATTCATTTGTATGATGCGT-3′). The recombinant polh-C containing a His-tag at the N-terminus was expressed by *E.col*i BL21 (DE3) cells at 37°C for 3 h. Thereafter, *E. coli* were collected by centrifugation (6,000 × *g* for 10 min) and sonicated for 20 min in 5% glycerol in PBS. The recombinant polh-C was solubilized with 1.5% sarcosine and purified using Ni-NTA beads (Qiagen). The binding beads were washed 3 times, 4 × Laemmli buffer was added, and then proteins were separated using 12.5% SDS-PAGE. The purified protein was remove from the polyacrylamide gel and injected into rabbit to produce the polyclonal antibodies.

Wild-type PmNV OBs were separated by SDS-PAGE and transferred to a polyvinylidene difluoride membrane (Amersham). The membrane was blocked in 5% skim milk at 4°C overnight and incubated with anti-polyhedrin antibody (1:10,000) at room temperature for 1 h. Anti-rabbit immunoglobulin G antibody (Santa cruz) conjugated to horseradish peroxidase (1:10,000) was used as the secondary antibody. A chemiluminescence system (Perkin Elmer) was used for detection.

### Nucleotide sequence accession number

The PmNV genome sequence was submitted to GenBank under accession number KJ184318.

### Availability of supporting data

The complete PmNV genome sequence has been submitted to GenBank (accession number KJ184318). The phylogenetic tree shown in Figure 2 has been deposited in TreeBase http://purl.org/phylo/treebase/phylows/study/TB2:S15937. All supporting data is included as additional files.

## Electronic supplementary material

Additional file 1: Table S1: Primers designed to amplify ~1500 nucleotides PCR fragments with 500-nucleotide overlaps covering the entire PmNV genome. (DOCX 55 KB)

Additional file 2: Table S2: Comparisons of two high-throughput results including deletions, insertions and nucleotide errors. (DOCX 17 KB)

Additional file 3: Table S3: Comparisons of three sequencing results. (DOCX 16 KB)

Additional file 4: Table S5: Structures of PmNV direct repeat sequences. (DOCX 18 KB)

Additional file 5: Table S4: Comparisons of different ORFs prediction results by DNAMAN, FGENESV0, GLIMMER3 and GeneMarkS software. (DOCX 14 KB)

Additional file 6: Figure S1: Gene parity plots comparing ORF content and order of PmNV with (A) HzNV-1 and (B) OrNV. ORFs present in only one of the compared genomes appear on the axis corresponding to the virus in which they are present. (PDF 94 KB)

Additional file 7: Figure S2: The conserved *helicase*and *pif-4*gene cluster on the genome of PmNV, HzNV-1, GbNV and OrNV (Figure adapted from Wang *et al.,* [68]. Nudivirus genomics and Phylogeny. In *Viral genomes –molecular structure, diversity, geneexpression mechanisms and host-virus interactions*(Chapter 2). Intech. ). ORFs are represented by arrows. The numbers above and below the arrows represent the sequential ordering of the ORFs on the respective viral genomes.The viral genomesare represented as bold lines with omitted genomic rangesindicated by dots. (PDF 174 KB)

Additional file 8: Table S6: Sequence accession numbers of the phylogenetic tree analysis and the conserved domain comparisons of IAPs. (PDF 116 KB)

Additional file 9: Figure S3: Topological structures of selected baculovirus and nudivirus IAPs (inhibitors of apoptosis). Black lines indicate the relative lengths of each amino acid sequence; long greenboxes represent BIR domains; yellow pentangles are RING domains. Most baculovirus IAPs contain two BIR domains in the N terminal and a RING domain in the C terminal. By contrast, nudiviruses IAPs tend to be more diverse. IAP accession numbers are listed in Additional file 8: Table S6. (PDF 103 KB)

Additional file 10: Figure S4: Micrographs of PmNVOBs from PmNV-infected hepatopancreatictissue that had beenhomogenized and lysed by 1/3 PBS.(A) Lysate of hepatopancreaswas lysed by 1/3 PBS. (B)-(E) OBs in the resuspended pellets and the supernatants after centrifugation at 4,000 × *g*for 10 min or 13,000 × *g*for 30 min as indicated. Scale bar: 50 μm. (PDF 223 KB)

## Abbreviations

PmNV: *Penaeus monodon* nudivirus
PL: Postlarval
AcMNPV: *Autographa californica* nucleopolyhedrovirus
OB: Occlusion body
MBV: Monodon baculovirus
ICTV: International committee on taxonomy of viruses
PemoNPV: *Penaeus monodon* nucleopolyhedrovirus
NGS: Next Generation Sequencing
hrs: Homology regions
HzNV-1: *Heliothis zea* nudivirus 1
HzNV-2: *Helicoverpa zea* nudivirus 2
OrNV: *Oryctes rhinoceros* nudivirus
GbNV: *Gryllus bimaculatus* nudivirus
MRNuV: *Macrobrachium* nudivirus
PvSNPV: *Penaeus vannamei* single nucleopolyhedrovirus.
