# Supplementary material for: The genome and occlusion bodies of marine Penaeus monodon nudivirus (PmNV, also known as MBV and PemoNPV) suggest that it should be assigned to a new nudivirus genus that is distinct from the terrestrial nudiviruses
Source: BMC Genomics. 2014 Jul 25;15(1):628. doi: 10.1186/1471-2164-15-628 (PMC4132918; doi:10.1186/1471-2164-15-628)
Supplement: Supplementary file 1 — Additional file 1: Table S1: Primers designed to amplify ~1500 nucleotides PCR fragments with 500-nucleotide overlaps covering the entire PmNV genome. (DOCX 55 KB) [file 12864_2014_6342_MOESM1_ESM.docx]

Table S1. Primers designed to amplify ~1500 nucleotides PCR fragments with 500-nucleotide overlaps covering the entire PmNV genome.

| Name | Start | Stop | Sequence (5'-3') |
| --- | --- | --- | --- |
| F1 | 31 | 54 | ATGGACGACCTTAGTGGAGATCAG |
| R1 | 1821 | 1798 | GTGGAAGTAGAAGAGCCTGAAGCA |
| F2 | 1050 | 1073 | TAGCAATACTTCTTCCCGCACACC |
| R2 | 2554 | 2535 | TGTGTGCCGCAGAACAAAGA |
| F3 | 2056 | 2079 | TGTCTGAAGACGTAGAGCTCATGC |
| R3 | 3578 | 3557 | GAAAGAGTTCCTGCCAGCCATA |
| F4 | 3090 | 3111 | CTATTCACCGGATCAAGCTCCA |
| R4 | 4594 | 4575 | AATGATCGGGCCTACGGAGG |
| F5 | 4005 | 4029 | AGAGCATACAGGTTGTCAGTGGTCG |
| R5 | 5522 | 5499 | CCAATGACCAGAAAGTACGCTCAG |
| F6 | 5069 | 5092 | TGAGGGAATCCATCCAAGTGTTGG |
| R6 | 6624 | 6601 | GCCAGTTGGAATTCGATGGTCGTA |
| F7 | 6004 | 6027 | GTCCGTTACGTCAACTCCAATTGG |
| R7 | 7561 | 7538 | GCACTTCGTGATAGCATCCTCGAT |
| F8 | 7052 | 7075 | CACTTAAGAATGTAGCCAAGGCCC |
| R8 | 8577 | 8556 | GCGATTTGCAGAGTTCTTGACG |
| F9 | 8385 | 8408 | TGGCCTACTTGGAGCATCATATGG |
| R9 | 9897 | 9876 | CATCGGGCATGGTGAACATATG |
| F10 | 9117 | 9138 | TGCTTACGCAATCGAACAGACC |
| R10 | 10829 | 10806 | GAAGGTAGAGTCGGAAGTGGTTGA |
| F11 | 10018 | 10041 | TTATGTTGGCAGATGGTGGCATCG |
| R11 | 11704 | 11681 | GGATGGCTCCGTACTATCAGACGA |
| F12 | 11074 | 11097 | TTGACAGTAGGTGCAGGAAGTTCC |
| R12 | 12678 | 12656 | GGACTAACGGTACCCATTGAAGG |
| F13 | 12006 | 12027 | TTCCCGATGGTTCATCCTTGAC |
| R13 | 13708 | 13685 | GGCCTTTAACTGGTTGACAGTGAC |
| F14 | 13059 | 13082 | TCTTCCCTTCGTCATCTGGAGTAC |
| R14 | 14585 | 14562 | CCACGCTGTTTGTAGATGCTGTAG |
| F15 | 14001 | 14020 | CAAAAGATGGGGCGGAATTG |
| R15 | 15761 | 15742 | GCAATGCAAACGGATCATGC |
| F16 | 15049 | 15072 | TATTCAGCATGCTGGTATCTGGGC |
| R16 | 16631 | 16610 | CTGGGCTGGCAGCAGTATTATT |
| F17 | 16003 | 16027 | GGACACGTCTCTACATGGATGTACC |

Table S1. continued.

| Name | Start | Stop | Sequence (5'-3') |
| --- | --- | --- | --- |
| R17 | 17502 | 17483 | ACGGGATATGCCATCGCAAC |
| F18 | 17094 | 17117 | CGTGAGTCACTCGAGTCTCTTCAA |
| R18 | 18685 | 18662 | GCATTTGGAGACTACCGACTCGTT |
| F19 | 18283 | 18304 | TTCGGTTTCGTCTCTCACTTCG |
| R19 | 19787 | 19768 | GCCGATTTGCTAGATGACGA |
| F20 | 19129 | 19152 | GAAATATCGTCGTTCTGAGGGGTC |
| R20 | 20720 | 20701 | TGGCGAGTGCAATTTTGTCC |
| F21 | 20160 | 20183 | GAAAGAGTGGATCTCGTCTTCAGC |
| R21 | 21986 | 21963 | CTGTCGTCGTCGTCAGTCTTATTG |
| F22 | 21049 | 21070 | AAGATTCGAGATGTGGCCAACG |
| R22 | 22624 | 22603 | GAACCCTTCCAAAAGCCAGAAG |
| F23 | 22068 | 22091 | TCCTGGGAAATCATGAGTATGCCC |
| R23 | 23623 | 23609 | CGCACACAGTTGGATCATTCTGGA |
| F24 | 23034 | 23057 | CCTTGGATATTCTCTTGGGCTTGG |
| R24 | 24635 | 24712 | GGGAACATTAGGGCAAGCGAATTC |
| F25 | 24378 | 24401 | TTGATGCTGCTCTGAGTAAAGCCC |
| R25 | 25917 | 25894 | GGTCCACTCTCAACCAGTTTCTTG |
| F26 | 25179 | 25199 | CTGAAATGGCACAGATGGCAC |
| R26 | 26696 | 26675 | CAAGGTCTGGTTGGAACGAAAC |
| F27 | 26215 | 26238 | TTACAGCTACATCATCCCTCTGGG |
| R27 | 27818 | 27795 | GCACCTTATCTGGCTGACCATAGA |
| F28 | 27119 | 27142 | TCTCTCTCAGACAAGATGGGGAAC |
| R28 | 28946 | 28926 | GCCGAAGGTTCACTTCAATGG |
| F29 | 28228 | 28252 | CAGATTCTTCACCCCTCTCTTCTCG |
| R29 | 29786 | 29763 | GATGGAAGAGACGCTAAAGGACAC |
| F30 | 29160 | 29181 | TGGAAGAAGCTGTCCGTGAACA |
| R30 | 30762 | 30739 | CGAGAAGCGGTGCAAAATCTAGGA |
| F31 | 30035 | 30059 | GGATCCCTTAGAACTGACACTCTCC |
| R31 | 31678 | 31658 | CGAGAAGAGGAAGCATTTCGC |
| F32 | 31086 | 31109 | ATCTGTTCGTTTGCTTCAGGTGCG |
| R32 | 32643 | 32624 | TGCTGCCTTGTTCGCATTGA |
| F33 | 32094 | 32117 | TCCTTGTAGCTACTCCTGGATGGT |
| R33 | 33739 | 33716 | GGAGTTGGGCTGGTTTTAGTAAGC |
| F34 | 33060 | 33083 | GTTGCACACGTAAGGCTACAGTTG |
| R34 | 34608 | 34585 | CGGCAGTGTACAAGAGCTTAAGTG |
| F35 | 34077 | 34100 | AGGTTTATTGTCCTGGCCAGAGAC |

Table S1. continued.

| Name | Start | Stop | Sequence (5'-3') |
| --- | --- | --- | --- |
| R35 | 35576 | 35557 | TTTGGCAGCCTACGACCTTG |
| F36 | 35295 | 35318 | TCCTCACTATCTCGAAGATCTGGC |
| R36 | 36853 | 36829 | GCATTGCATATCTATGAAACCAAGC |
| F37 | 36086 | 36109 | TTGGGTTATTTGCCTCTGCTGTCG |
| R37 | 37664 | 37623 | GCACAATTGGTCGGATAACCCT |
| F38 | 37291 | 37315 | AATATTCAGAAGCTTTCCGATACCG |
| R38 | 38831 | 38808 | GGCAAAGAGTATAGCATCTCAGGG |
| F39 | 381193 | 38142 | ACTGAGGAACTAGAGCGAGGGTTT |
| R39 | 39642 | 39618 | CAGTAGACTCTGAGAGCGCAGATCA |
| F40 | 39060 | 39083 | TGCGATAAACTTCCTGGATCTGCC |
| R40 | 40597 | 40576 | GGGGCTGTATGGTCGTTTAAGC |
| F41 | 40222 | 40243 | CTTGATACTCAAAGATGCGGCG |
| R41 | 41946 | 41926 | TCTCCCCTCAACAACGTGTGA |
| F42 | 41063 | 41086 | GTTAGGGGTGAATGTAGTGTTGCC |
| R42 | 42570 | 42546 | GCAGTGACCTACTCTTGGTTAGTGG |
| F43 | 42082 | 42105 | CACCCCAAAACTGCAATTGTAGGC |
| R43 | 43621 | 43597 | GGGAAGGTCAGTTAGATAGCCTCGA |
| F44 | 43102 | 43122 | CTCTGTTAAAACAGGCCCCGA |
| R44 | 44668 | 44649 | GCGCCAATGGAATTTCCAAC |
| F45 | 44053 | 44074 | CTATTGGAACATTGGCATCGGC |
| R45 | 45693 | 45670 | GGGCTATGCAAACATCAACTGCCT |
| F46 | 45068 | 45087 | TGCAATCTTTGGCATGCCTG |
| R46 | 46575 | 46552 | CAAGCTATTACGAGCCCTTTGTGC |
| F47 | 46043 | 46064 | GGGGGCCAAGTTATACTGTTCG |
| R47 | 47547 | 47525 | GTGGCTGACTCGTGAGTTCTTCA |
| F48 | 47237 | 47260 | CATTGGTATACAGCACACAGAGCC |
| R48 | 48950 | 48929 | CACTTATGCCATATCCCCGAGA |
| F49 | 48073 | 48096 | CCAAGGAGGTGTATGTAAAACCCC |
| R49 | 49588 | 49567 | AGGGTGTGCCTATTTCCAAGCT |
| F50 | 49043 | 49067 | TCCCACAAATATTGATTCCATTCTG |
| R50 | 50587 | 50563 | CAGTGCTACCAAACTAGGAAAACGA |
| F51 | 50473 | 50494 | ATTACCCCATGGGAAGTCGTTG |
| R51 | 51975 | 51954 | ATCCTCTTCCTGCTGCACTCAA |
| F52 | 51388 | 51412 | AAGGCCATTAGAGGCTTCAACCCCT |
| R52 | 53013 | 52990 | GTGCCATGCACACAGGAGTATCAT |
| F53 | 52043 | 52062 | GAAGCCCAGGTTGCAGCATT |

Table S1. continued.

| Name | Start | Stop | Sequence (5'-3') |
| --- | --- | --- | --- |
| R53 | 53609 | 53586 | GGGCTTTCTGGCATCTGATACTGA |
| F54 | 53182 | 53205 | TGGTAGAAAGTGAAGATGGGTGGC |
| R54 | 54683 | 54663 | CCAATACCGTCACCGGATCAC |
| F55 | 54236 | 54259 | GTTGTGTCAGCGTTGCTGTTAGAG |
| R55 | 55762 | 55739 | CCTGGCTCTTTGAATGTGATTGGG |
| F56 | 55058 | 55082 | AGAACAGAGTACCTGACCCCTGACC |
| R56 | 56725 | 56701 | CGGATGCAAGTCCTACTACAGTACG |
| F57 | 56276 | 56297 | AATTCTCACAAGCAGCCAGCTC |
| R57 | 57804 | 57781 | CCTCCTTGCGAGACCCATATTTCA |
| F58 | 57245 | 57268 | AGCCATAAACGTGACCCAAGATCC |
| R58 | 58794 | 58771 | CGACCCAACGTAATTATCACCTCC |
| F59 | 58043 | 58064 | TTCGCGTAGCTTCACGTACACG |
| R59 | 59737 | 58715 | TGTTCGACAGTATGGCTCTCCAC |
| F60 | 59352 | 59375 | CGTCTCTATTGATGGCACCTGTTG |
| R60 | 60948 | 60927 | CCAGCCAAAAGGTAAATACGCC |
| F61 | 60490 | 60511 | TTGTGGTTGAGGGGTCATTGAG |
| R61 | 61995 | 61971 | GGGCATCCACTAGTCATTTTCATCT |
| F62 | 61081 | 61105 | ACAATTTGAATACGAGCGAATCTTG |
| R62 | 62813 | 61790 | CAAGTCGTATTGCCACTACCTTCG |
| F63 | 62262 | 62283 | TGATGAGCGGTAAAAGTGGTCG |
| R63 | 63786 | 63762 | CACTGAAAAAGAACGACACGATTGT |
| F64 | 63043 | 63066 | CAACCACCAGTACTGAGGAGGAGT |
| R64 | 64714 | 64691 | CTCCACGACGAGTTTGCTCTACAA |
| F65 | 64235 | 64258 | TTGCAGCAGGCAGTATGAATTCGG |
| R65 | 65853 | 65830 | CGATTGCAGGATCCATCATACCCA |
| F66 | 65057 | 65079 | GTCCAGGTCCAGGTCTCGTTAAT |
| R66 | 66670 | 66647 | GCTGGTAACACACCTTCGTTTGCA |
| F67 | 66133 | 66156 | TGAGAGCTCTGTACTTTCGAAGGC |
| R67 | 67896 | 67873 | GGTCACTGGGTTCTTGATGCCATA |
| F68 | 67177 | 67198 | ATGGTGGACCATTATTCGGCTG |
| R68 | 68680 | 68659 | CTCACGAGATTGCCATGGCTAT |
| F69 | 68193 | 68216 | AGCACCATTTGTACTCATCTCGGC |
| R69 | 69692 | 69673 | ACGATGAATCGGCACTCCAA |
| F70 | 69162 | 69185 | TACTTTGGGGGTGTAACAGATGGG |
| R70 | 70690 | 70669 | GCTTGGCAAAGAGCTTATGCGA |
| F71 | 70204 | 70227 | TCGATTTGGTCGTATTGTGGTCGC |

Table S1. continued.

| Name | Start | Stop | Sequence (5'-3') |
| --- | --- | --- | --- |
| R71 | 72027 | 72006 | CGAAGTCCAAGAAGCCATCGAA |
| F72 | 71054 | 71075 | ATGGAACAGGTGAATGGCAGTC |
| R72 | 72610 | 72591 | TGCTGCCATTGGCCTGACTG |
| F73 | 72150 | 72173 | TGTAAGAACCCCCTTGTTTCCAGG |
| R73 | 73649 | 73630 | GTGGATCTGCCGTGGAATGA |
| F74 | 73071 | 73095 | GAGCACTACAATAGGGTAGCGTTGC |
| R74 | 74582 | 74559 | GCGAGCCAGAAAGTCTAATTGCAC |
| F75 | 74113 | 74136 | TCAAACTGGACGATGACTCTGACG |
| R75 | 75730 | 75707 | CCAAGAATTCATCGGGCCAGAGAT |
| F76 | 75167 | 75190 | TGAGTGTAGACGATTGAGTGAGGC |
| R76 | 76674 | 76650 | TGTACGCCTGGTCTGTATCAGCTAC |
| F77 | 76108 | 76131 | CCTTTGCTCAGAATATCTCCCTGG |
| R77 | 77608 | 77589 | ATCCAGCATCTGCCGCATGT |
| F78 | 77112 | 77133 | CTTTGGCATAACGGACGAAGTC |
| R78 | 78703 | 78680 | CAGAGGAAGAAGAGGCATGCTACA |
| F79 | 78403 | 78426 | TTCAAAAACGGCTTCGGTGTAGGC |
| R79 | 79907 | 79886 | CCCAGACCTCAGAAGCACGAGT |
| F80 | 79122 | 79143 | AATGACTGACCTCAGTTTGCGC |
| R80 | 80682 | 80661 | GGCCAACATGATGCTTTCTCCA |
| F81 | 80100 | 80123 | TCGAGTATGGAACATCTGCAACGC |
| R81 | 81602 | 81583 | GCCGGAATTGTGAGCATCAA |
| F82 | 81100 | 81123 | CCTATATCCACCCAAAAGTCAGGC |
| R82 | 82603 | 82582 | CGGGTCGATCATAAGGCTTTGT |
| F83 | 82063 | 82084 | GGGTTACACGAAAGCTTCATGC |
| R83 | 83754 | 83731 | GAACAGTAGATCACAGGGGATTGG |
| F84 | 83212 | 83232 | GAAGTCCATGAGCCCACTCGA |
| R84 | 84816 | 84793 | GACCATCTTCTTCTTGCCTCACCT |
| F85 | 84287 | 84306 | TAACCCAAATGGGAGTGCCA |
| R85 | 85871 | 85850 | CACGGTTGGCAGATAAACACCA |
| F86 | 85320 | 85343 | CAATACCCCCTGAAAGAGCTTACC |
| R86 | 87055 | 87036 | TGGCGCCATCCAACCTATTT |
| F87 | 86221 | 86244 | CTGCCCACATTGCAAGAGTAGATG |
| R87 | 87736 | 87713 | TCAGATAAGACTTGCCAGTACCCC |
| F88 | 87249 | 87270 | TGCAGGGTTAAAGGTCACTGGT |
| R88 | 88801 | 88778 | GAGTGAGCCCTTGAATGAAGTGGT |
| F89 | 88516 | 88540 | GCTAAGTTTCTACCGTACCTGCTCC |

Table S1. continued.

| Name | Start | Stop | Sequence (5'-3') |
| --- | --- | --- | --- |
| R89 | 90035 | 90012 | CGATATACGTTACTCGGCTGGCAA |
| F90 | 89244 | 89268 | CCAGTACCAGTAGCAACTCAAGTGC |
| R90 | 90786 | 90763 | GTTGTCGTCTCCAGCTGTTCAATC |
| F91 | 90316 | 90340 | CAGTACTCCCATAGTGACTGCCAGT |
| R91 | 92187 | 92163 | CGTAGTACTGATAGGACGGGCCATT |
| F92 | 91226 | 91249 | AAAGCATTGAGTGCCATAGAGCCC |
| R92 | 93078 | 93055 | CGCTCTCCTGTAGCTTGTACGAAT |
| F93 | 92251 | 92274 | CGAGAACTGGGATATGCTGAGAAC |
| R93 | 93761 | 93737 | TGAAGAGTTTCGACAAAATGCAAAT |
| F94 | 93266 | 93289 | AATGCACGCATTCCTAGACCTAGC |
| R94 | 94776 | 94753 | GAGAGCGTTTGGTAAAACTGACCC |
| F95 | 94246 | 94269 | CATCTCCACCAGTATTGGGTCTTG |
| R95 | 95749 | 95728 | CGTAGTAAAATCTGCCGGGATG |
| F96 | 95477 | 95498 | TGGAACGCTCTTCGATATCTGC |
| R96 | 97056 | 97033 | GACGAGAGCGCTGCATAATTACTC |
| F97 | 96370 | 96393 | AATTCTTCCGCTTGAGCCACTACC |
| R97 | 97875 | 97854 | GTGTGCATTGCATCGCCATCTA |
| F98 | 97442 | 97463 | TTATCAAACCGACAGCAGGCTG |
| R98 | 98950 | 98927 | GGTGGATTATTCAATGGGACCCTC |
| F99 | 98391 | 98412 | CCACGATGATCTGGGGATCTAA |
| R99 | 99898 | 99874 | CATTTTTATGAGTGCGAAGAGAAGC |
| F100 | 99224 | 99247 | GATAAGAACATCGCTACCGAGCTG |
| R100 | 100765 | 100742 | GCGATAGAGTTGGCTCCATATGCA |
| F101 | 100665 | 100688 | GTATCCTGTTCCGGTAGCTTTCTG |
| R101 | 102170 | 102146 | CTCAGTACAAGGCAAATTGCAAAGT |
| F102 | 101255 | 101278 | CTATAGCCTCCGACTTTGAAGGTG |
| R102 | 103090 | 103069 | GTTGGAGTTTCGCGTATCAACG |
| F103 | 102361 | 102382 | GTCGTACGCGTTGCTGGCACTA |
| R103 | 103866 | 103843 | CCGTGTACAGTGTGGATGGTATTG |
| F104 | 103458 | 103481 | TCTCTAGCAACAGAAGCGACCTTG |
| R104 | 105073 | 105052 | GGTATAGCAAGCGAGCGCTTTT |
| F105 | 104399 | 104422 | GATGCTAGCTCGTGTATATTCCCC |
| R105 | 106123 | 106100 | CGACTTCCCAGTACGATGTCTGAA |
| F106 | 105551 | 105574 | GGAGAGTATTTGGTGATCGCTCAG |
| R106 | 107117 | 107094 | CGGTACCTTAGCAGGGATAATGGT |
| F107 | 106212 | 106235 | CACAAGTCTTGGGAATTTGCAATT |

Table S1. continued.

| Name | Start | Stop | Sequence (5'-3') |
| --- | --- | --- | --- |
| R107 | 107866 | 107842 | TCGAAATGCCAATACAATATCGATT |
| F108 | 107212 | 107235 | CTGAATCTCCAGAGACCTTTCGAC |
| R108 | 108748 | 108728 | GGGTGGTGCGCAATCATTGTA |
| F109 | 108248 | 108271 | CTAAAGCCGTCTCAAGACATCAGC |
| R109 | 109856 | 109833 | CCGAACTAGCGAGTGACGAATATG |
| F110 | 109371 | 109394 | TATCATCGTCATCCACTTCAGGGC |
| R110 | 110901 | 110878 | GGGGTGTAGGAATCGAAGAAACTC |
| F111 | 110318 | 110341 | CATTTCTTAGCCTTGCCCGAAACG |
| R111 | 111843 | 111822 | CGGCTTGTTTTGCTTCGGGAAA |
| F112 | 111287 | 111308 | CGAATTGTGGGAACTCGTGAGC |
| R112 | 112811 | 112790 | CCAAGACGGTAATGATTGCCAC |
| F113 | 112218 | 112241 | ATACATCGTTCACCCTGAGAGCTG |
| R113 | 113720 | 113699 | ATAGCACCAGCGAGTCCTCTGC |
| F114 | 113303 | 113326 | TTAGCGACATCCAATGCCACTTCG |
| R114 | 114915 | 114892 | GGAATTGGACTCCATCACTGGTTC |
| F115 | 114482 | 114503 | GACATCCCGCCCTGAATCCCAA |
| R115 | 116122 | 116099 | GCCAGAATGACGTTGTTCATGCAG |
| F116 | 115219 | 115240 | CACGTTCTTCAGAATTGGTCCG |
| R116 | 116766 | 116742 | CCTGCTGTCTGGTGTAACAACTAGC |
| F117 | 116221 | 116244 | GGAAAAAGTACTACACAGACCCGG |
| R117 | 117764 | 117741 | CCAACGGTCTTTACGGGAAGTGTA |
| F118 | 117211 | 117231 | CTTTATCGGGTCTGCAGTGGG |
| R118 | 118766 | 118746 | CCCAGTGCTACAATGGCTGCA |
| F119 | 118225 | 118248 | AGGCTTATGGTGCATACCACAGAG |
| R119 | 119306 | 119283 | GCCGGTACAATATTGCCAGGTTGA |
| F120 | 118870 | 118900 | GATGCAAATATGACTTTTTTAGGTAATGCT |
| R120 | 731 | 712 | CCGTTAGCATTGGCACCCAA |
| 2-F-1 | 1713 | 1732 | CATCATCATCATCTTGCTGC |
| 3-F-1 | 2714 | 2731 | GAGAGCCAAGGTCTTATC |
| 4-F-1 | 3683 | 3700 | GCTGAAGTGGATGTTGTC |
| 5-F-1 | 4670 | 4688 | GTCCTTGAAACTCACTACG |
| 6-F-1 | 5804 | 5821 | CTCTCTCTCCACACATAC |
| 7-F-1 | 6665 | 6683 | AAGGTACGGAGATCATAAC |
| 8-F-1 | 7785 | 7801 | CTCTGCAGCTTACGTCC |
| 9-F-1 | 9124 | 9142 | GCAATCGAACAGACCAATC |
| 10-F-1 | 9802 | 9819 | CTGATGTATGCTGTGCTC |

Table S1. continued.

| Name | Start | Stop | Sequence (5'-3') |
| --- | --- | --- | --- |
| 11-F-1 | 10807 | 10825 | CAACCACTTCCGACTCTAC |
| 12-F-1 | 11742 | 11761 | CATCCTCCACTTTCAATTCG |
| 13-F-1 | 12782 | 12745 | CCATTGAATGCCTGACCT |
| 14-F-1 | 13789 | 13806 | CGATGCTCGGTTTAGAGA |
| 15-F-1 | 14722 | 14739 | CGGTTCGTTTCAGGGTAA |
| 16-F-1 | 15787 | 15804 | GCTAAACTCTCAGCATCC |
| 17-F-1 | 16792 | 16809 | GGCCATGTCAAAGTAAGG |
| 18-F-1 | 17731 | 17749 | GGATTGCAGGATTACTTCG |
| 19-F-1 | 19066 | 19086 | GGCTACTAGTTCAATACACCC |
| 20-F-1 | 19820 | 19837 | GATCTTGCCGCTTTCCTA |
| 21-F-1 | 20887 | 20904 | GTAGCCAAACCGGTTAAG |
| 23-F-1 | 22780 | 22797 | GATAGTGGCCGGTTGTAT |
| 24-F-1 | 23797 | 23814 | GAAGCTTCCCGTCATGTT |
| 25-F-1 | 25100 | 25119 | GAATATCTGAACATCTGGCC |
| 25-F-1 | 25179 | 25196 | CTGAAATGGCACAGATGG |
| 26-F-2 | 25862 | 25882 | GGAGGTACTAATGAAGATGGC |
| 27-F-1 | 26885 | 26905 | GACCTAAATACAGTCCTGGTG |
| 28-F-1 | 27836 | 27855 | GAAGCATGTAAGACAGGAAC |
| 28-R-1 | 28220 | 28200 | GGATACCTCTGATAATGATGC |
| 29-F-1 | 28934 | 28951 | GTGAACCTTCGGCCTTCC |
| 30-F-1 | 29845 | 29862 | GCGAGAGCATGACATTTG |
| 30-R-1 | 30059 | 30040 | GGAGAGTGTCAGTTCTAAGG |
| 31-F-1 | 30769 | 30786 | GTTTAGCCAGCACTACAC |
| 31-R-1 | 30907 | 30890 | GATCCTACGGTTTTCTCG |
| 31-R-2 | 31018 | 31000 | GAAGAGTCTGTAGTGGAAG |
| 32-F-1 | 31793 | 31810 | CTTGGCTTTGGCAGTCAC |
| 32-R-1 | 31978 | 31970 | CACCAGGCCTTGTCATTG |
| 33-F-1 | 32760 | 32778 | CCCTCAAAGTACGTACAAG |
| 33-R-1 | 33052 | 33033 | GCGAAACCAGTATTTACCAG |
| 34-R-1 | 34152 | 34135 | GGTGATAAGAAGCGCTGG |
| 35-F-1 | 34773 | 34792 | CATAAACTACATGCGCCATC |
| 36-F-1 | 36027 | 36048 | GCATCAAAGACAGAATCCATAG |
| 37-F-1 | 36760 | 36780 | CCTTGCTGAATTCTTCATCTG |
| 38-R-1 | 38137 | 38120 | CTCGCTCTAGTTCCTCAG |
| 39-F-1 | 38808 | 38829 | CCCTGAGATGCTATACTCTTTG |
| 40-F-1 | 39781 | 39800 | GAATGATGGGCATGATATGG |

Table S1. continued.

| Name | Start | Stop | Sequence (5'-3') |
| --- | --- | --- | --- |
| 42-R-1 | 41887 | 41870 | GGTGTGCTAAGGATTCGA |
| 43-R-1 | 42791 | 42774 | GCCTTTAGCCGAGCATAT |
| 43-R-1 | 42812 | 42795 | GACTCGAGGGTGTAAAGA |
| 44-R-1 | 43826 | 43809 | CATTTACCGTTGGCACGA |
| 45-F-1 | 44754 | 44772 | GATGATGATTCGTTGCCAG |
| 46-R-1 | 45754 | 45735 | GAAGTCATCCCATCTATGCG |
| 47-F-1 | 46768 | 46785 | CCCAATAAAAGCGCTCCA |
| 48-F-1 | 48017 | 48034 | TGACCAGAGCCACTTTGA |
| 49-F-1 | 48930 | 48947 | CTCGGGGATATGGCATAA |
| 50-F-1 | 49853 | 49870 | CCAAGTCGAACACTCCAT |
| 51-R-1 | 51355 | 51335 | CATATTGAATAATGGCAAGCC |
| 52-F-1 | 52082 | 52099 | CTGAACTTTCCATGCTTG |
| 53-F-1 | 52720 | 52740 | CTGATACCGAAGGTTATTGTC |
| 54-F-1 | 53798 | 53817 | GACAGAAGAGTTTAAGGTTG |
| 55-F-1 | 54909 | 54929 | GGTTTCTTGGGCTTGAGATTC |
| 56-R-1 | 56100 | 56079 | GTACATTGCTATAACCTTAGTG |
| 57-F-1 | 56947 | 56967 | CGCGATATATTACAAGTGATG |
| 58-F-1 | 57922 | 57941 | CCAAGCGAGATATAGAAGAG |
| 59-R-1 | 59208 | 59187 | CCAATAATCTGAATGTCCTCCC |
| 60-F-1 | 59974 | 59994 | GAGGATATTTTAGAAGCAGTC |
| 61-F-1 | 61088 | 61105 | GAATACGAGCGAATCTTG |
| 62-R-1 | 62182 | 62161 | GGAATTGCTCTTATCTACAGAC |
| 63-F-1 | 62957 | 62978 | GACTGCTTTATTACCAAGAGTG |
| 64-R-1 | 64006 | 63989 | CAGACACAATCACCGAAG |
| 65-F-1 | 64890 | 64908 | GAAGAACTCGAAACTGTGG |
| 66-F-1 | 65689 | 65706 | CCAATCTCACACTCGATC |
| 67-F-1 | 66762 | 66779 | GGGCACAAGTCTCACAAG |
| 68-F-1 | 67878 | 67895 | CATCAAGAACCCAGTGAC |
| 69-F-1 | 68879 | 68898 | CCATGTGAAGTTTGTCGTAG |
| 70-F-1 | 69859 | 69876 | GACCCTACAATTCAGACG |
| 71-F-1 | 70889 | 70906 | TCGATTTATGCTATGCCC |
| 72-F-1 | 71737 | 71760 | GTTATAGTTGCTCTCATTATGTAC |
| 73-R-1 | 73025 | 73008 | GGTATTGGTGGTGATAGG |
| 74-F-1 | 71747 | 73765 | GCATCTACCCTGCTACGAG |
| 75-F-1 | 74777 | 74795 | GTTGTGTGGATTGTGCGTC |
| 75-R-1 | 75087 | 75069 | CTAGACAGTGCGCTTCAAC |

Table S1. continued.

| Name | Start | Stop | Sequence (5'-3') |
| --- | --- | --- | --- |
| 76-F-1 | 75882 | 75900 | GATGTGTTGAATTTGGTCG |
| 77-F-1 | 76812 | 76831 | GGTTTGGGTTTCATTGGTTG |
| 78-F-1 | 77740 | 77758 | CCCATATAAACAGTAACGG |
| 79-F-1 | 79013 | 79035 | GGAAATATCCAACATCATACGAG |
| 80-F-1 | 79767 | 79786 | GATGTTGGTTCGGGTAACGG |
| 81-F-1 | 80757 | 80778 | GGTTCTCAATATCTCTTCATCG |
| 82-F-1 | 81791 | 81810 | GATCATTCAATCTGTGTTCG |
| 83-F-1 | 82777 | 82798 | CCGATAGAAAAACCTACAGATC |
| 83-R-1 | 83032 | 83011 | GCTTATCAATTGGCTTTGTAGG |
| 84-F-1 | 83878 | 83897 | GACCCTAATAGTCAGTATGG |
| 84-R-1 | 84196 | 84177 | CACTGATTACGCACCATCTC |
| 85-F-1 | 84927 | 84945 | CATGCTGAAGAATCGTTTG |
| 86-F-1 | 85989 | 86008 | TATGCAGATCAAACACAATG |
| 86-R-1 | 86358 | 86337 | CTTCCATGGCAAATCGATAATC |
| 88-F-1 | 87973 | 87991 | TCGTTGGAGTTTGACTTAC |
| 88-R-1 | 88134 | 88115 | ACCTCTATGCAATTTAGACG |
| 89-R-1 | 89356 | 89335 | AAGAGATTCTATCGTATCATCG |
| 89-R-2 | 89382 | 89364 | GGTTCTAAGGTTTCTGCTC |
| 90-F-1 | 89963 | 89986 | CCATATCTTACCGATCAGTATACC |
| 91-R-1 | 91498 | 91478 | CGCTCTTGTCAATTCTTCTAG |
| 92-F-1 | 91945 | 91964 | CGATAAGTCGTTTGAGATGG |
| 92-R-1 | 92360 | 92339 | CTATGATATCAGCCATACGTTG |
| 93-F-1 | 93048 | 93069 | GTCTCGTATTCGTACAAGCTAC |
| 94-F-1 | 93951 | 93972 | GTGAAAGGCTCGATTATCTTAC |
| 95-R-1 | 95137 | 95119 | GCAGCTTCCAGAAATACAC |
| 96-F-1 | 96177 | 96195 | CCGTATGCAATCACAGTTC |
| 97-F-1 | 97053 | 97070 | CGTCAAAGAGTACAACCC |
| 98-F-1 | 98066 | 98085 | GCATGGACTCAACGATATAG |
| 99-F-1 | 99036 | 99054 | GAGAACATGACTCGTTTCC |
| 100-F-1 | 99867 | 99884 | CGTCTTAGCTTCTCTTCG |
| 101-F-1 | 101356 | 101378 | GTACTTGTATCCATATCTTCCAG |
| 102-F-1 | 101889 | 101914 | GGTAGTTGTAGTAGAATTATTCATCG |
| 102-R-1 | 102353 | 102334 | CACATCGAGTGCAATAAAGC |
| 103-F-1 | 103098 | 103119 | GCAGCCAACTGTTCTTTAAATC |
| 104-F-1 | 104084 | 104105 | GAGTAAATACTCGACCACTCTC |
| 105-F-1 | 105137 | 105158 | GACTAACTTGCAGAATTCTACC |

Table S1. continued.

| Name | Start | Stop | Sequence (5'-3') |
| --- | --- | --- | --- |
| 106-F-1 | 106268 | 106289 | CGAAAGAACGGACATTTCTATC |
| 106-R-1 | 106390 | 106372 | GCGTATGACATGACTCAAG |
| 107-F-1 | 106967 | 106987 | GCAGTGCCCTATGAATTATAC |
| 108-F-1 | 108004 | 108027 | GAATCCGCGAGTTAAATTACTTTC |
| 109-F-1 | 108885 | 108902 | GATACAACAGCGAACCAG |
| 110-F-1 | 110044 | 110062 | TCCATAAAGATCGCCAATC |
| 111-F-1 | 110957 | 110977 | CATGTATTGCAATTCTTCTGG |
| 112-F-1 | 111950 | 111970 | GACTTTCCAAATAAGCACTCC |
| 113-F-1 | 112906 | 112923 | CAGAGCAGCATACCGATC |
| 114-F-1 | 114017 | 114033 | GAACTAGATCGGACCAG |
| 115-F-1 | 115181 | 115198 | TGTAGCATCTGACTCTGC |
| 116-F-1 | 115903 | 115922 | TATGCCTGGTAAGTATGTTC |
| 117-F-1 | 116845 | 116862 | CACCCTTTACACCATTCG |
| 117-R-1 | 117102 | 117083 | CACCTCAAGATATCACTAGC |
| 118-F-1 | 117912 | 117931 | CGTAAACTACTTCCACATTG |
