# Supplementary material for: The genome and occlusion bodies of marine Penaeus monodon nudivirus (PmNV, also known as MBV and PemoNPV) suggest that it should be assigned to a new nudivirus genus that is distinct from the terrestrial nudiviruses
Source: BMC Genomics. 2014 Jul 25;15(1):628. doi: 10.1186/1471-2164-15-628 (PMC4132918; doi:10.1186/1471-2164-15-628)
Supplement: Supplementary file 2 — Additional file 2: Table S2: Comparisons of two high-throughput results including deletions, insertions and nucleotide errors. (DOCX 17 KB) [file 12864_2014_6342_MOESM2_ESM.docx]

Table S2. Comparisons of two high-throughput results including deletions, insertions and nucleotide errors.

| **Genome location** | **1^st^ sequencing**  **(119,428 bp )** | **2^nd^ sequencing**  **(119,128 bp )** | **ORF** |
| --- | --- | --- | --- |
| 76,197 | 1 nt deletion (G) | ✓ | 🗴 |
| 114,098 | 1 nt insertion (G) | ✓ | 🗴 |
| 98,197 | ✓ | nucleotide error (T🡪C) | - |
| 98,215 | ✓ | nucleotide error (A🡪G) | - |
| 98,219 | ✓ | nucleotide error (A🡪G) | - |
| 18,025 | 24 nt deletion TTGGTTTTGTAATTCATTAGTTTG | ✓ | Pif-2  (in frame) |
| 33,098 | 18 nt deletion  AGGGAGAAAGAGAGAGAG | 467 nt deletion | 🗴 |
| 82,780 | 168 nt deletion | 89 nt deletion | Zonadhesin  239🡪294 aa |

“✓” = nucleotide sequence is correct

“🗴” = sequence error is not in predicted ORF

“-“ = sequence error does not effect to the ORF prediction
