# Supplementary material for: The genome and occlusion bodies of marine Penaeus monodon nudivirus (PmNV, also known as MBV and PemoNPV) suggest that it should be assigned to a new nudivirus genus that is distinct from the terrestrial nudiviruses
Source: BMC Genomics. 2014 Jul 25;15(1):628. doi: 10.1186/1471-2164-15-628 (PMC4132918; doi:10.1186/1471-2164-15-628)
Supplement: Supplementary file 3 — Additional file 3: Table S3: Comparisons of three sequencing results. (DOCX 16 KB) [file 12864_2014_6342_MOESM3_ESM.docx]

Table S3. Comparisons of three sequencing results

|  | **1^st^ high-throughput sequencing** | **2^nd^ high-throughput sequencing** | **Sanger sequencing** |
| --- | --- | --- | --- |
| Raw data | F1 : 1,871,268  R2 : 1,871,268 | F1 : 5,371,230  R2 : 5,371,230 | - |
| Total counts | 3,742,536 | 10,742,460 | - |
| Mapping counts | 1,039,052 | 906,658 | - |
| Mapping rate | 27.76% | 8.44% | - |
| Single contiguous contig (nt) | 119,426 | 119,128 | 119,638 |
