# Supplementary material for: The genome and occlusion bodies of marine Penaeus monodon nudivirus (PmNV, also known as MBV and PemoNPV) suggest that it should be assigned to a new nudivirus genus that is distinct from the terrestrial nudiviruses
Source: BMC Genomics. 2014 Jul 25;15(1):628. doi: 10.1186/1471-2164-15-628 (PMC4132918; doi:10.1186/1471-2164-15-628)
Supplement: Supplementary file 4 — Additional file 4: Table S5: Structures of PmNV direct repeat sequences. (DOCX 18 KB) [file 12864_2014_6342_MOESM4_ESM.docx]

Table S5. Structures of PmNV direct repeat sequences

| Type | Genome location | Repeat unit | Copy Number | Consensus pattern | A+T % | Total length (bp) |
| --- | --- | --- | --- | --- | --- | --- |
| *dr*1 | 17972-18074 | 24 | 4.3 | GTTTGTTGGTTTTGCAATTCATTA | 71 | 113 |
| *dr*2 | 33271-33311 | 20 | 2.0 | CATGACTCAGCACTTTTTAG | 60 | 41 |
| *dr*3 | 33426-33465 | 20 | 2.0 | TATATAATTTTTTTATTATA | 100 | 40 |
| *dr*4 | 33602-33686 | 35 | 2.4 | AAAAGCTAGAACATAAAAGTTAGAATGATAGATAT | 80 | 85 |
| *dr*5 | 33849-33962 | 16 | 7.3 | GAGAAAGAGAGAGAGG | 50 | 114 |
| *dr*6 | 40040-40065 | 3 | 8.7 | TGA | 67 | 26 |
| *dr*7 | 64942-64972 | 18 | 2.3 | AGGTCTAGGTCCAGGTCC | 39 | 42 |
| *dr*8 | 75402-75532 | 36 | 3.8 | ATTAAATCTTACAATACATCAGTAAACATTACAATC | 78 | 131 |
| *dr*9 | 82699-82982 | 42 | 6.8 | GAGAAGCCTACAGATCCAATTGATAAACCTACGAAACCGATA | 60 | 284 |
| *dr*10 | 110679-110711 | 16 | 2.0 | ATATTATATATTAATTT | 100 | 33 |
