# Supplementary material for: The genome and occlusion bodies of marine Penaeus monodon nudivirus (PmNV, also known as MBV and PemoNPV) suggest that it should be assigned to a new nudivirus genus that is distinct from the terrestrial nudiviruses
Source: BMC Genomics. 2014 Jul 25;15(1):628. doi: 10.1186/1471-2164-15-628 (PMC4132918; doi:10.1186/1471-2164-15-628)
Supplement: Supplementary file 5 — Additional file 5: Table S4: Comparisons of different ORFs prediction results by DNAMAN, FGENESV0, GLIMMER3 and GeneMarkS software. (DOCX 14 KB) [file 12864_2014_6342_MOESM5_ESM.docx]

Table S4. Comparisons of different ORFs prediction results by DNAMAN, FGENESV0, GLIMMER3 and GeneMarkS software.

| Software | Number of ORFs | Plus strand | Minus strand | Parameter |
| --- | --- | --- | --- | --- |
| DNAMAN | 216 | 115 | 101 | Coding more than 50 aa |
| FGENESV0 | 104 | 53 | 51 | Standard code and circular genome type |
| GLIMMER3 | 104 | 54 | 50 | Standard code and circular genome type |
| GeneMarkS | 97 | 50 | 47 | Intronless eukaryotic-virus |
