# Supplementary material for: The genome and occlusion bodies of marine Penaeus monodon nudivirus (PmNV, also known as MBV and PemoNPV) suggest that it should be assigned to a new nudivirus genus that is distinct from the terrestrial nudiviruses
Source: BMC Genomics. 2014 Jul 25;15(1):628. doi: 10.1186/1471-2164-15-628 (PMC4132918; doi:10.1186/1471-2164-15-628)
Supplement: Supplementary file 6 — Additional file 6: Figure S1: Gene parity plots comparing ORF content and order of PmNV with (A) HzNV-1 and (B) OrNV. ORFs present in only one of the compared genomes appear on the axis corresponding to the virus in which they are present. (PDF 94 KB) [file 12864_2014_6342_MOESM6_ESM.pdf]

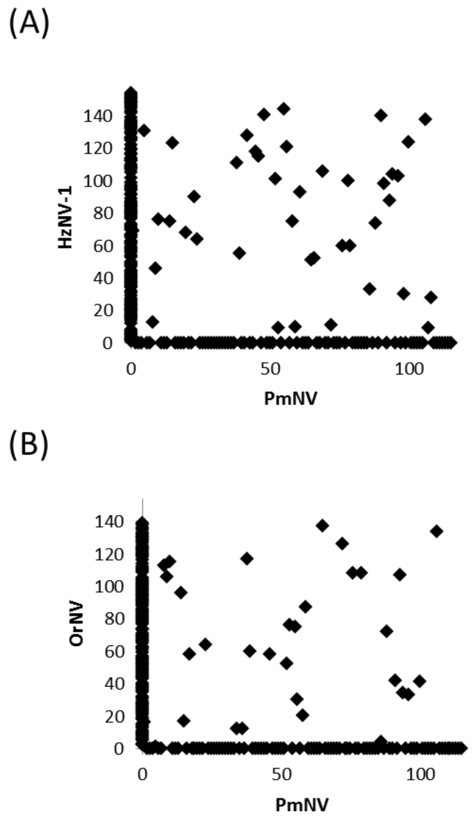

Fig. S1. Gene parity plots comparing ORF content and order of PmNV with (A) HzNV-1 and (B) OrNV. ORFs present in only one of the compared genomes appear on the axis corresponding to the virus in which they are present.
