# Supplementary material for: The genome and occlusion bodies of marine Penaeus monodon nudivirus (PmNV, also known as MBV and PemoNPV) suggest that it should be assigned to a new nudivirus genus that is distinct from the terrestrial nudiviruses
Source: BMC Genomics. 2014 Jul 25;15(1):628. doi: 10.1186/1471-2164-15-628 (PMC4132918; doi:10.1186/1471-2164-15-628)
Supplement: Supplementary file 7 — Additional file 7: Figure S2: The conserved helicaseand pif-4gene cluster on the genome of PmNV, HzNV-1, GbNV and OrNV (Figure adapted from Wang et al., [68]. Nudivirus genomics and Phylogeny. In Viral genomes –molecular structure, diversity, geneexpression mechanisms and host-virus interactions(Chapter 2). Intech. ). ORFs are represented by arrows. The numbers above and below the arrows represent the sequential ordering of the ORFs on the respective viral genomes.The viral genomesare represented as bold lines with omitted genomic rangesindicated by dots. (PDF 174 KB) [file 12864_2014_6342_MOESM7_ESM.pdf]

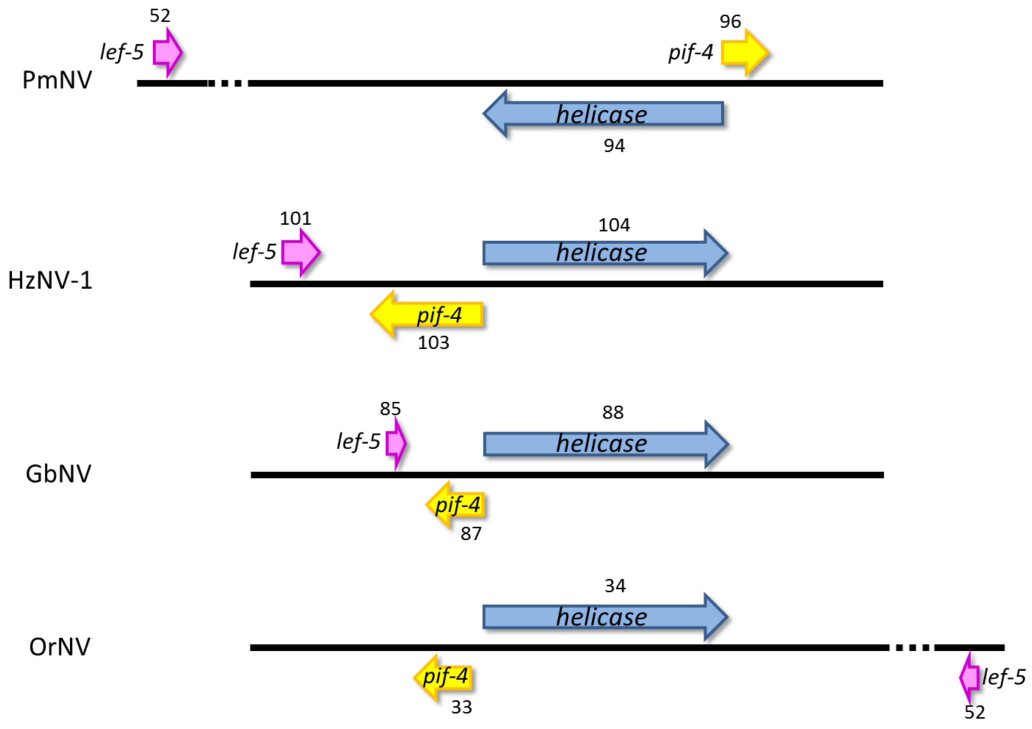

Fig. S2. The conserved *helicase* and *pif-4* gene cluster on the genome of PmNV, HzNV-1, GbNV and OrNV (Figure adapted from Wang *et al.*, 2012. Nudivirus genomics and Phylogeny. In *Viral genomes – molecular structure, diversity, gene expression mechanisms and host-virus interactions* (Chapter 2). Intech.). ORFs are represented by arrows. The numbers above and below the arrows represent the sequential ordering of the ORFs on the respective viral genomes. The viral genomes are represented as bold lines with omitted genomic ranges indicated by dots.
