# Supplementary material for: The genome and occlusion bodies of marine Penaeus monodon nudivirus (PmNV, also known as MBV and PemoNPV) suggest that it should be assigned to a new nudivirus genus that is distinct from the terrestrial nudiviruses
Source: BMC Genomics. 2014 Jul 25;15(1):628. doi: 10.1186/1471-2164-15-628 (PMC4132918; doi:10.1186/1471-2164-15-628)
Supplement: Supplementary file 8 — Additional file 8: Table S6: Sequence accession numbers of the phylogenetic tree analysis and the conserved domain comparisons of IAPs. (PDF 116 KB) [file 12864_2014_6342_MOESM8_ESM.pdf]

Table S6. Sequence accession numbers of the phylogenetic tree analysis and the conserved domain comparisons of IAPs.

Gene name / Virus	*dna pol*	*helicase*	*lef-4*	*lef-5*	*lef-8*	*lef-9*	*p47*	*p74*	*pif-1*	*pif-2*	*pif-3*	*iap*
**AcMNPV**	NP_054095	AAA67907	NP_054120	NP_054129	NP_054079	NP_054092	NP_054069	NP_054168	NP_054149	NP_054051	NP_054145	NP_054056
**BmNPV**	NP_047469	CAA71181	NP_047490	NP_047500	NP_047454	NP_047466	NP_047446	NP_047536	NP_047516	AFN08941	AFN09024	-
**LyxyMNPV**	YP_003517819	YP_003517833	YP_003517829	YP_003517836	YP_003517788	YP_003517801	YP_003517784	YP_003517764	YP_003517888	YP_003517878	YP_003517852	-
**CpGV**	NP_148895	NP_148874	NP_148879	NP_148871	NP_148915	NP_148901	NP_148852	NP_148844	NP_148859	NP_148832	NP_148819	AAB39098
**AgseGV**	YP_006243	YP_006264	YP_006259	YP_529743	YP_006226	YP_006237	YP_006284	YP_006288	YP_006279	YP_006301	YP_006315	-
**SpliGV**	YP_001257052	YP_001257031	YP_001257037	YP_001257027	YP_001257072	YP_001257058	YP_001257009	YP_001257007	YP_001257015	YP_001256990	YP_001256978	-
**NeleNPV**	YP_025217	YP_025258	YP_025259	YP_025255	YP_025279	YP_025237	YP_025246	YP_025247	YP_025277	YP_025252	YP_025266	YP_025208
**NeseNPV**	YP_025135	YP_025168	YP_025169	YP_025165	YP_025188	YP_025147	YP_025156	YP_025157	YP_025186	YP_025162	YP_025176	-
**NeabNPV**	YP_667860	YP_667909	YP_667910	YP_667906	YP_667931	YP_667887	YP_667897	YP_667898	YP_667927	YP_667903	YP_667917	-
**CuniNPV**	NP_203396	NP_203394	NP_203400	NP_203391	NP_203330	NP_203363	NP_203377	NP_203378	NP_203333	NP_203342	NP_203350	-
**HzNV-1**	AAM45757	AAN04398	AAN04392	AAN04395	AAN04384	AAN04369	AAN04369	AAM45758	AAN04349	AAN04416	AAN04382	AAN04430
**HzNV-2**	YP_004956766	YP_004956786	YP_004956791	YP_004956788	YP_004956799	YP_004956811	YP_004956811	YP_004956854	YP_004956830	YP_004956774	YP_004956801	YP_004956760
**OrNV**	ABF93350	YP_002321345	YP_002321353	YP_002321363	YP_002321375	YP_002321407	YP_002321331	YP_002321437	YP_002321371	YP_002321328	YP_002321418	YP_002321445
**GbNV**	YP_001111279	YP_001111355	YP_001111363	YP_001111352	YP_001111316	YP_001111291	YP_001111336	YP_001111312	YP_001111319	YP_001111333	YP_001111270	YP_001111365
**MdSGHV**	YP_001883329	-	-	-	-	-	-	YP_001883367	YP_001883357	YP_001883417	YP_001883434	-
**GpSGHV**	YP_001687027	-	-	-	-	-	-	YP_001686949	YP_001687050	YP_001687001	YP_001687049	-
**ASFV**	-	NP_042799	-	-	-	-	-	-	-	-	-	-
**MRNuV**	-	-	-	-	-	-	-	-	-	-	-	AFP33715
