# Supplementary material for: The genome and occlusion bodies of marine Penaeus monodon nudivirus (PmNV, also known as MBV and PemoNPV) suggest that it should be assigned to a new nudivirus genus that is distinct from the terrestrial nudiviruses
Source: BMC Genomics. 2014 Jul 25;15(1):628. doi: 10.1186/1471-2164-15-628 (PMC4132918; doi:10.1186/1471-2164-15-628)
Supplement: Supplementary file 9 — Additional file 9: Figure S3: Topological structures of selected baculovirus and nudivirus IAPs (inhibitors of apoptosis). Black lines indicate the relative lengths of each amino acid sequence; long greenboxes represent BIR domains; yellow pentangles are RING domains. Most baculovirus IAPs contain two BIR domains in the N terminal and a RING domain in the C terminal. By contrast, nudiviruses IAPs tend to be more diverse. IAP accession numbers are listed in Additional file 8: Table S6. (PDF 103 KB) [file 12864_2014_6342_MOESM9_ESM.pdf]

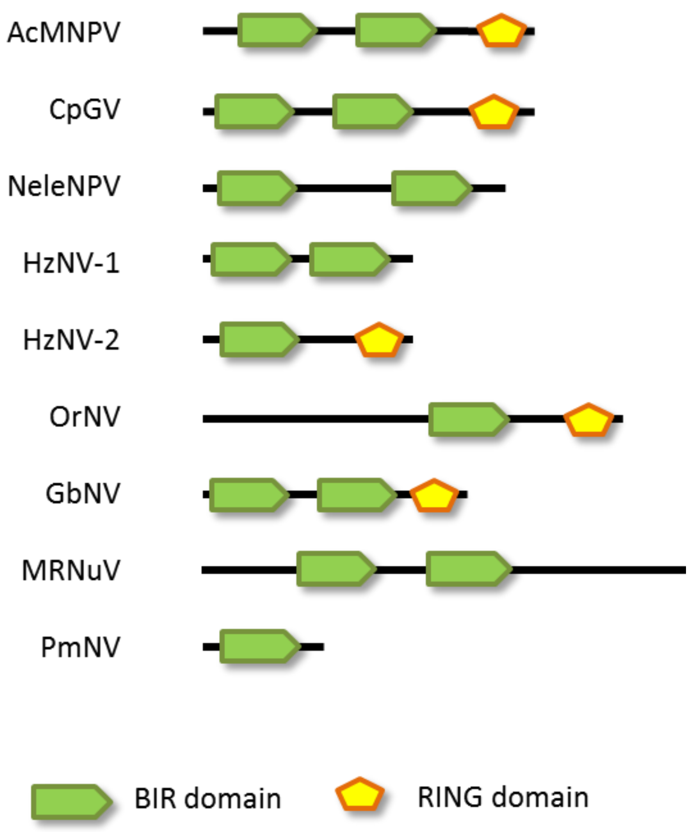

Fig. S3. Topological structures of selected baculovirus and nudivirus IAPs (inhibitors of apoptosis). Black lines indicate the relative lengths of each amino acid sequence; long green boxes represent BIR domains; yellow pentangles are RING domains. Most baculovirus IAPs contain two BIR domains in the N terminal and a RING domain in the C terminal. By contrast, nudiviruses IAPs tend to be more diverse. IAP accession numbers are listed in Table S6.
