# Supplementary material for: The genome and occlusion bodies of marine Penaeus monodon nudivirus (PmNV, also known as MBV and PemoNPV) suggest that it should be assigned to a new nudivirus genus that is distinct from the terrestrial nudiviruses
Source: BMC Genomics. 2014 Jul 25;15(1):628. doi: 10.1186/1471-2164-15-628 (PMC4132918; doi:10.1186/1471-2164-15-628)
Supplement: Supplementary file 10 — Additional file 10: Figure S4: Micrographs of PmNVOBs from PmNV-infected hepatopancreatictissue that had beenhomogenized and lysed by 1/3 PBS.(A) Lysate of hepatopancreaswas lysed by 1/3 PBS. (B)-(E) OBs in the resuspended pellets and the supernatants after centrifugation at 4,000 × gfor 10 min or 13,000 × gfor 30 min as indicated. Scale bar: 50 μm. (PDF 223 KB) [file 12864_2014_6342_MOESM10_ESM.pdf]

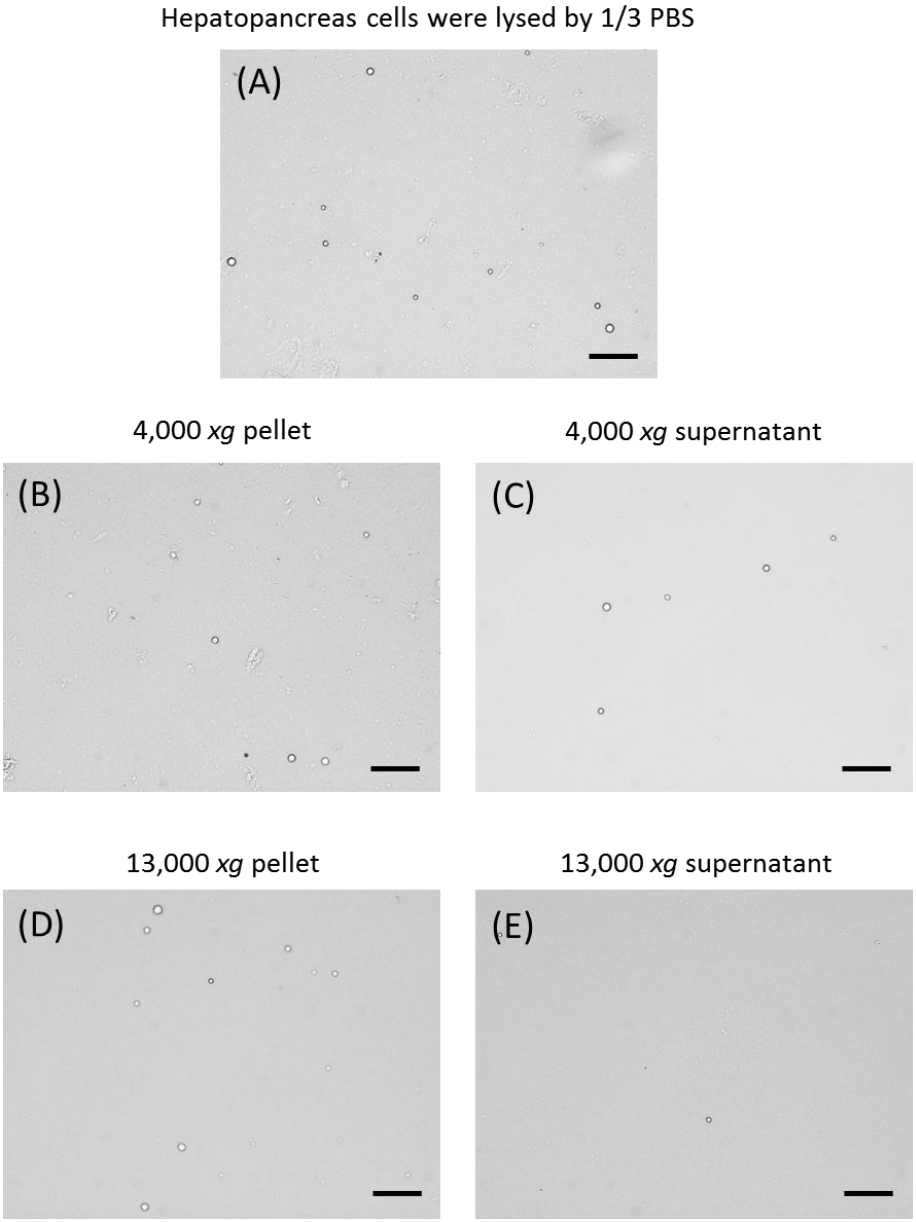

Fig. S4. Micrographs of PmNV OBs from PmNV-infected hepatopancreatic tissue that had been homogenized and lysed by 1/3 PBS. (A) Lysate of hepatopancreas was lysed by 1/3 PBS. (B) - (E) OBs in the resuspended pellets and the supernatants after centrifugation at 4,000 ×*g* for 10 min or 13,000 ×*g* for 30 min as indicated. Scale bar: 50 µm.
